# Changes in DNA Methylation in *Arabidopsis thaliana* Plants Exposed Over Multiple Generations to Gamma Radiation

**DOI:** 10.3389/fpls.2021.611783

**Published:** 2021-03-31

**Authors:** Pol Laanen, Eline Saenen, Mohamed Mysara, Jorden Van de Walle, May Van Hees, Robin Nauts, Filip Van Nieuwerburgh, Stefan Voorspoels, Griet Jacobs, Ann Cuypers, Nele Horemans

**Affiliations:** ^1^Biosphere Impact Studies, Belgian Nuclear Research Centre (SCK CEN), Mol, Belgium; ^2^Centre for Environmental Research, Hasselt University, Diepenbeek, Belgium; ^3^Laboratory of Pharmaceutical Biotechnology, Ghent University, Ghent, Belgium; ^4^NXTGNT, Ghent University, Ghent, Belgium; ^5^Vlaamse Instelling voor Technologisch Onderzoek, VITO, Mol, Belgium

**Keywords:** ionising radiation, DNA methylation, multigenerational, adaptation, epigenetics, whole genome bisulfite sequencing (WGBS), transposable elements

## Abstract

Previous studies have found indications that exposure to ionising radiation (IR) results in DNA methylation changes in plants. However, this phenomenon is yet to be studied across multiple generations. Furthermore, the exact role of these changes in the IR-induced plant response is still far from understood. Here, we study the effect of gamma radiation on DNA methylation and its effect across generations in young *Arabidopsis* plants. A multigenerational set-up was used in which three generations (Parent, generation 1, and generation 2) of 7-day old *Arabidopsis thaliana* plants were exposed to either of the different radiation treatments (30, 60, 110, or 430 mGy/h) or to natural background radiation (control condition) for 14 days. The parental generation consisted of previously non-exposed plants, whereas generation 1 and generation 2 plants had already received a similar irradiation in the previous one or two generations, respectively. Directly after exposure the entire methylomes were analysed with UPLC-MS/MS to measure whole genome methylation levels. Whole genome bisulfite sequencing was used to identify differentially methylated regions (DMRs), including their methylation context in the three generations and this for three different radiation conditions (control, 30 mGy/h, and 110 mGy/h). Both intra- and intergenerational comparisons of the genes and transposable elements associated with the DMRs were made. Taking the methylation context into account, the highest number of changes were found for cytosines followed directly by guanine (CG methylation), whereas only limited changes in CHG methylation occurred and no changes in CHH methylation were observed. A clear increase in IR-induced DMRs was seen over the three generations that were exposed to the lowest dose rate, where generation 2 had a markedly higher number of DMRs than the previous two generations (Parent and generation 1). Counterintuitively, we did not see significant differences in the plants exposed to the highest dose rate. A large number of DMRs associated with transposable elements were found, the majority of them being hypermethylated, likely leading to more genetic stability. Next to that, a significant number of DMRs were associated with genes (either in their promoter-associated region or gene body). A functional analysis of these genes showed an enrichment for genes related to development as well as various stress responses, including DNA repair, RNA splicing, and (a)biotic stress responses. These observations indicate a role of DNA methylation in the regulation of these genes in response to IR exposure and shows a possible role for epigenetics in plant adaptation to IR over multiple generations.

## Introduction

In the last decade, the role of epigenetics in stress responses of plants, as well as their effect on gene expression has gained more attention. Epigenetic modifications, such as DNA methylation, small interfering RNA (siRNA), and histone modifications, can alter the way chromatin is packaged and can be accessed (Boyko and Kovalchuk, 2008). As such, a change in epigenetic marks can have a great impact on overall genome stability and gene expression. For example, in *Arabidopsis thaliana* DNA repeats and transposable elements (TEs) are highly correlated with cytosine methylation, which is essential for genome integrity (Vaillant and Paszkowski, 2007; Brautigam and Cronk, 2018). Stress conditions can lead to epigenetic modifications and thereby alter genome stability as well as gene expression and thus epigenetic modifications might play a role in adaptation to these stressors (Horemans et al., 2019; Schmid et al., 2018). Alterations in DNA methylation, for example, have been implicated in plant responses to several stresses (i.e., salinity, pathogen, UV, drought, water, heat stress) (Dowen et al., 2012; Sahu et al., 2013; Kinoshita and Seki, 2014).

More recently, the role of epigenetics in plant responses to ionising radiation (IR) is gaining interest. Low levels of natural background IR are present everywhere on Earth as a result of cosmic radiation and naturally occurring radionuclides in the Earth’s crust. However, human activities have caused a significant increase in these dose rates and this can potentially have a negative impact on the environment (e.g., the nuclear accidents in Chernobyl and Fukushima). The IR stress responses in plants has been mainly studied on a phenotypical, physiological, biochemical, and genetic level. Some effects are still under debate such as the change in flowering induction, either by resulting in earlier or later flowering (Sax, 1955; Gunckel, 1957; Daly and Thompson, 1975; Kovalchuk et al., 2007; Hwang et al., 2016; Kryvokhyzha et al., 2018), or the effect on seed germination (Kumagai et al., 2000). Ionising radiation can have severe damaging biological effects either directly, by damaging biomolecules including DNA, or indirectly, by the production of reactive oxygen species (ROS) in the organism. These ROS are products of the radiolysis of, amongst others, water and these ROS will, when not scavenged by the plant’s antioxidative defence system, lead to oxidative stress and damage to e.g., DNA molecules. As a result, DNA damage occurs often in organisms exposed to IR (West et al., 2000; Esnault et al., 2010; Dona et al., 2013; Biermans et al., 2015; Van Hoeck et al., 2017). In order to protect itself from the harmful effects of IR, processes such as oxidative stress response (i.e., increase in antioxidants) and DNA repair mechanisms will be called upon by the organism (Esnault et al., 2010; Biermans et al., 2015; van de Walle et al., 2016; Einor et al., 2016; Volkova et al., 2017). Previous research has shown that IR also affects the epigenome, of which DNA methylation has been the most studied (for an overview see Horemans et al., 2019). Pine trees from sites contaminated by the Chernobyl accident showed a dose-rate dependent increase in global DNA methylation (Kovalchuk et al., 2003; Volkova et al., 2018). A similar observation was made in soybeans that have grown in the Chernobyl exclusion zone for seven generations (Georgieva et al., 2017). However, results from *A. thaliana* sampled along radiation gradients in the exclusion zone showed some contradicting findings, demonstrating either an overall hypermethylation or hypomethylation (Kovalchuk et al., 2004; Horemans et al., 2018).

It has been established that DNA methylation of TEs is a tool to regulate their activity and it is therefore common to find high levels of DNA methylation located in these regions (Rabinowicz et al., 2003; Ikeda and Nishimura, 2015). Additionally, TE relocation has been shown to be activated by IR amongst other stressors in *A. thaliana* (Wang et al., 2016). These TEs play an important role in genetic evolution as they can result in significant genetic changes by inversion, deletion, inactivating or activating genes (Muñoz-López and García-Pérez, 2010). IR-induced hypermethylation can be seen as a defence response to prevent genome instability by prohibiting reshuffling of genetic material, such as TEs (Kovalchuk et al., 2004; Boyko et al., 2007; Horemans et al., 2018; Volkova et al., 2018).

In addition to its importance in gene regulation, DNA methylation’s heritable character has recently gained interest for its potential role in acclimation and/or adaptation over generations to environmental stress conditions (Verhoeven et al., 2010; Boyko and Kovalchuk, 2011; Hauser et al., 2011; Mirouze and Paszkowski, 2011). Acclimation occurring in one generation as a method of overcoming changes in the environment or stressors has been widely studied in plants (de Azevedo Neto et al., 2005; Chinnusamy and Zhu, 2009; Chinnusamy et al., 2010). For instance, increased UV resistance was achieved by priming plants to low levels of chronic UV exposure (Hideg et al., 2013). This led to changes in antioxidant levels which enabled plants to cope better with increased oxidative stress induced by a second UV exposure. Similar indications of acclimation have been seen in plants in response to salinity and IR (Munns and Gilliham, 2015; Van Hoeck et al., 2017). Although the exact nature of priming is still not fully understood, previous studies have shown that epigenetics, including DNA methylation, and TEs might play a role in this priming mechanism (Espinas et al., 2016; Negin and Moshelion, 2020; Turgut-Kara et al., 2020). Adaptation over one or more generations to stress also remains under debate (Pecinka et al., 2009; Rasmann et al., 2012; Moller and Mousseau, 2016), nonetheless, a number of reports have demonstrated transgenerational adaptive stress responses in plants (Verhoeven and van Gurp, 2012; Suter and Widmer, 2013; Groot et al., 2016). However, with these studies it is important to keep in mind the difference between transgenerational studies, which explore the inherited effects over generations after exposure to stress in the first generation, and multigenerational studies, which explore the inherited effects over generations that are all exposed to a similar stress factor in each generation. Work on plant survival and reproduction in the Chernobyl exclusion zone, the Fukushima affected area, as well as studies done in lab conditions continue to contribute to the uncovering of a potential adaptation to IR exposure (Zaka, 2002; Geras’kin et al., 2005; Danchenko et al., 2009; Klubicova et al., 2012; Pozolotina et al., 2012; Rashydov and Hajduch, 2015; Georgieva et al., 2017; Kryvokhyzha et al., 2018). As mentioned, heritable epigenetic changes, such as DNA methylation, might play an important role in the adaptive responses to environmental stress (Schmid et al., 2018; Horemans et al., 2019).

To investigate the potential role of DNA methylation in plant responses to IR, exposure within one generation and over generations was performed in this study. It is hypothesised that IR induces a different cytosine DNA methylation profile in plants that are exposed compared to unexposed plants. Secondly, it is expected that plants with a different history in IR exposure will respond differently, in respect of DNA methylation, compared to plants that did not receive any prior IR exposure. In order to study this, we exposed three generations of *A. thaliana* plants [Parent (P0), Generation 1 (S1), Generation 2 (S2)] to five different dose rate conditions (natural background radiation (γ_0_), 30 mGy/h (γ_30_), 60 mGy/h (γ_60_), 110 mGy/h (γ_110_), and 430 mGy/h (γ_430_)) in a multigenerational experiment. First, the entire methylomes were analysed with UPLC-MS/MS to measure whole genome methylation levels. Secondly, whole genome bisulfite sequencing (WGBS) was used to identify differentially methylated regions (DMRs), including their methylation context. Based on this data, (1) both an intra- and intergenerational comparisons of the genes and TEs associated with the DMRs were made across the gamma radiation exposure conditions; and (2) a gene ontology enrichment was performed to discover the processes that might be regulated by IR-induced DNA methylation.

## Materials and Methods

### Plant Material and Gamma Treatment

**FIGURE 1:**
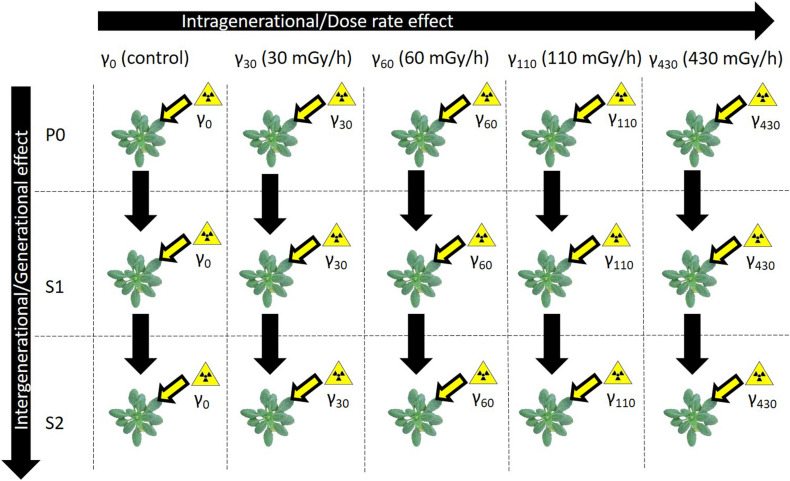
Schematic overview of the experimental set-up. Three generations of *A. thaliana* plants [Parent (P0), Generation 1 (S1), Generation 2 (S2)] were exposed to five different gamma dose rate conditions [natural background radiation (γ_0_), 30 mGy/h (γ_30_), 60 mGy/h (γ_60_), 110 mGy/h (γ_110_), and 430 mGy/h (γ_430_)]. S1 came from a previously exposed generation and S2 came from a line with two previously exposed generations.

In order to synchronise germination, *A. thaliana* (Columbia) seeds were vernalised on moist filter paper during 3 days at 4°C. The seeds of three different generations with a different irradiation background (2 weeks exposure to either γ_0_ = natural background radiation (control), γ_30_ = 30 mGy/h, γ_60_ = 60 mGy/h, γ_110_ = 110 mGy/h, or γ_430_ = 430 mGy/h) were used; P0 seeds originated from our standard seed stock and had never been irradiated, S1 seeds were harvested from the previously irradiated P0 plants and S2 seeds were harvested from previously irradiated S1 plants. This resulted in plants that had no previous history of irradiation (P0) and plants that already underwent the same gamma radiation treatments in one (S1) or two (S2) previous generations (Figure 1). Subsequently, the seeds were grown according to Vanhoudt et al. (2014) on cut-off plugs from 1.5 mL polyethylene centrifuge tubes filled with a Hoagland solution that was solidified with 0.6% agar and grown hydroponically in a growth cabinet (Snijders Scientific, Microclima 1000E) under a 14 h photoperiod (photosynthetically active radiation (PAR) of 200 μmol m^–2^ s^–1^ at the leaf level) with 65% humidity and a day/night temperature of 22°C/18°C. Roots were aerated during the entire course of the experiment and Hoagland solution was refreshed twice a week. When plants were 7 days old, their most vulnerable life stage for irradiation (Biermans et al., 2015), they were transferred to the irradiation unit of SCK CEN where they were exposed to gamma radiation during 14 days coming from a panoramical ^137^Cs-source. They were exposed to different dose rates (γ_30_ = 30 mGy/h, γ_60_ = 60 mGy/h, γ_110_ = 110 mGy/h, or γ_430_ = 430 mGy/h) of gamma radiation. These dose rate conditions were chosen based on previous experiments performed in our group. Under these conditions, *A. thaliana* plants exhibited radiation-induced biochemical and physiological changes, yet were still able to recover and produce viable following generations (Vanhoudt et al., 2010, 2014; Biermans et al., 2015; van de Walle et al., 2016). For each condition 2 containers containing 36 plants each, were used. After 14 days they received a total dose of, respectively, 7, 13, 29, and 156 Gy. During the irradiation period, plants were grown at 24°C and light was supplied by LED lights for 14 h photoperiodic period with a PAR of 200 μmol m^–2^ s^–1^ at the leaf level. Control plants were grown in a separate chamber at the same temperature and light conditions. After 14 days of irradiation fresh weight of plants was measured and the plant rosettes were harvested by snap-freezing them in liquid nitrogen and stored at −80°C until further analysis. Different treatments are indicated with a generation identifier and a treatment identifier. For example, P0γ_60_ refers to plants of the P0 generation that were exposed to the second gamma treatment (60 mGy/h), while S2γ_430_ refers to plants exposed to the highest dose rate (430 mGy/h) treatment in the S2 generation.

### DNA Extraction

Frozen plant samples (50–100 mg) were ground using a Mixer Mill MM 400 (Retsch) for 3 min at 30 Hz prior to the extractions. DNA was extracted from the ground material using Zymo ZR Plant/Seed DNA MicroPrep^TM^ kit according to the manufacturers’ instructions. The DNA quantity and integrity were determined spectrophotometrically at 230, 260, and 280 nm (Nanodrop, Isogen Life Science, De Meern, The Netherlands) and via gel electrophoresis (Bioanalyser, Agilent Technologies, Santa Clara, CA, United States), respectively.

### Global Methylation

The overall 5 methylcytosine (5mC) percentage was determined via UPLC, using five biological replicates of each (generational and dose rate) condition. One μg of extracted DNA was digested for 2 h at 37°C using the DNA Degradase Plus protocol according to Zymo Research Corporation (United States) which allows for a quick generation of single nucleotides from total DNA. Concentrations of 2′-deoxycytidine (dC) and 5-methyl-2′-deoxycytidine (5mdC) were measured with an Acquity Ultra Performance Liquid Chromatography (UPLC) system (Waters, Milford, MA, United States) coupled to a PDA detector (Waters, Milford, MA, United States) and a Micromass Quattro Premier XE mass spectrometer (Waters, United States). Chromatograms were analysed using Masslynx software v4.1 (Waters, Milford, MA, United States). Levels of dC and 5mdC were calculated based on the corresponding standard curves. The relative content of 5mdC was expressed as a percentage (%5mdC) with respect to the total amount of cytosine (dC + 5mdC). Several quality control measures were in place during the analysis. From a home-extracted control *A. thaliana* DNA pool several samples were used to monitor the method precision. Further, control standards and method blanks were analysed. Duplicate analysis of samples was performed whenever possible.

The statistical analysis of the global DNA methylation levels was performed using the open source software package R (R i386 3.1.0, R Foundation for Statistical Computing, Vienna, Austria). The normal distribution and homoscedasticity of our data were tested with a Shapiro-Wilk and Bartlett test, respectively. A one-way ANOVA test was applied to results from one generation or one treatment to identify any statistical differences between treatments and generations, respectively. When significant differences (*p* < 0.05) were found, a Tukey HSD test was applied to identify the specific differences between groups.

### Bisulfite Sequencing

Three different treatments (γ_0_ = control, γ_30_ = 30 mGy/h, γ_110_ = 110 mGy/h) per generation (P0, S1, S2) were selected for sequencing. This resulted in a total of nine different conditions with five biological replicates for each condition. Concentration of the extracted DNA was measured using the “Quant-it Picogreen dsDNA assay kit” (Life Technologies, Grand Island, NY, United States). Subsequently, 400–600 ng of gDNA was fragmented to 300 bp using the Covaris S2 focused-ultrasonicator (Covaris, Woburn, Massachusetts, United States). The size of the fragmented DNA was checked on a High sensitivity DNA chip (Agilent Technologies, Santa Clara, CA, United States). Library preparation with NEBNext Ultra II DNA library prep kit (New England Biolabs, Ipswich, MA, United States) was performed using methylated adapters, according to the manufacturer’s protocol. Size selection on a 2% EX Agarose E-Gel (Thermo Fisher Scientific, MA, United States) was performed on the resulting library, making a 300–1,000 bp gel cut followed by a purification with the Gel DNA recovery kit (Zymo Research, Irvine, CA, United States). Bisulfite conversion was performed with the EZ DNA Methylation Gold kit (Zymo Research, Irvine, CA, United States) according to the manufacturer’s protocol, followed by an additional purification with AMPure XP beads (Beckman Coulter, Brea, CA, United States) (beads:sample ratio of 5:1). An enrichment PCR was performed with KAPA Hifi hotstart Uracil + Ready mix (Kapa Biosystems, Wilmington, MA, United States) in a 13 cycles PCR reaction, followed by a purification with AMPure XP beads (Beckman Coulter, Brea, CA, United States) (beads:sample ratio of 1:1). Libraries were quantified by qPCR, according to Illumina’s protocol ‘Sequencing Library qPCR Quantification protocol guide’, version February 2011. A High sensitivity DNA chip (Agilent Technologies, Santa Clara, CA, US) was used to control the library’s size distribution and quality. Sequencing was performed on 2 high throughput Illumina NextSeq 500 flow cells generating PE2 × 75 bp reads. The flowcells were clustered with 2.3 pM library and 15% Phix control library.

### Differentially Methylated Region Assignment and Annotation

CLC Genomics Workbench 20.0^1^ was used to analyse the data. The paired reads were mapped to the reference genome (TAIR10.31)^2^ with “*Map Bisulfite reads to reference*” module, using non-directional approach (applying the default parameters). Differentially methylated regions were assigned using “*Call methylation level”* module by doing all pairwise comparison within the same generation or within the same treatment dose (resulting in 18 sets of DMRs identified for the different comparisons). The default parameters were applied (while specifying the minimum high-confidence site-coverage = 5 and minimum number of samples = 3), reporting the methylation levels for CG, CHG, and CHH contexts separately. The *p*-values produced from ANOVA statistical modelling where corrected using Benjamini Hochberg approach (using *p.adjust* in R v 3.5.0). Annotation to the nearest genes was added to each of the DMRs using “*Annotate by nearby gene*” module, using reference genome’s genes set. Additionally, the annotation to the nearest transposable elements (TEs) was added with *closest* module in bedtools package, using the “TAIR10 transposable elements” data set (downloaded on 18/03/2020) from the TAIR website^2^.

### Differentially Methylated Region Filtering and Functional Analysis

The filtering criteria to find DMRs associated with either gene regions (promoter or gene body) or TEs, were a *p* ≤ 0.05 and at least 20% difference in their methylation levels (referred to as methylation difference). This cut-off was chosen in order to ensure a definite methylation difference was being studied. Here, the methylation difference is calculated by comparing either a higher dose rate with a lower dose rate (i.e., S1γ_0_ vs. S1γ_30_), or by comparing an older generation with a younger on (i.e., P0γ_30_ vs. S1γ_30_). A hypermethylated DMR in P0γ_30_ vs. S1γ_30_ means that the region in S1γ_30_ has a higher methylation level compared to that of P0γ_30_.

For the filtering in DMRs associated with promoter regions, we chose to filter for DMRs with a distance to the nearest gene of 2 kbp ≤ X > 0. For those associated with gene bodies the filter was set at a distance of 0 to the nearest gene. DMRs associated with TEs were found by filtering for a distance of 0 to the nearest TE. For the functional analysis of the genes with DMRs, either in their promoter regions or their gene bodies, a gene ontology (GO) term enrichment was done using Metascape (Zhou Y. et al., 2019). For the analysis of overlapping genes in our selected comparisons, we used an online Venn diagram tool^3^ where we used the genes per comparison result as input.

## Results

### At a Global Methylation Level the First Offspring Generation Showed the Highest Radiation Response

**FIGURE 2:**
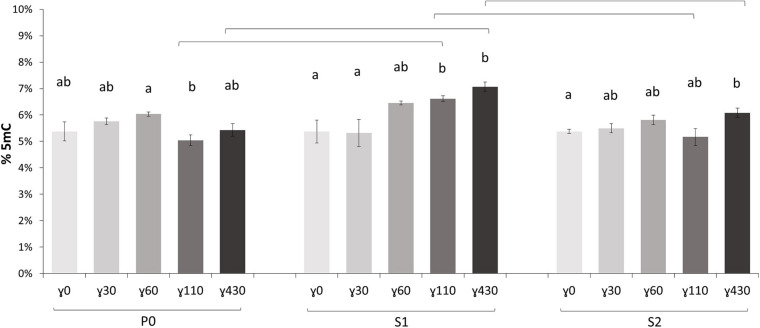
The global methylation percentage, determined with UPLC-MS/MS, of all generations [P0 (Parent generation), S1 (generation 1), S2 (generation 2)] of *A. thaliana* plants (γ_30_ = 30 mGy/h, γ_60_ = 60 mGy/h, γ_110_ = 110 mGy/h, and γ_430_ = 430 mGy/h). Values are represented relative to P0γ_0_ (effective values P0γ_0_ = 5.38%, S1γ_0_ = 4.88%, S2γ_0_ = 6.03%). Small letters indicate significant dose rate dependent differences (*p* < 0.05) within generations, brackets indicate significant (*p* < 0.05) differences at the same dose rate between generations. Measurements are an average ± SE of 5 biological replicates.

In the parent generation (P0) no significant dose rate dependent effects were seen on global methylation level (Figure 2). In the first generation (S1), however, dose rate dependent differences in comparison with control conditions were observed at the two highest dose rates γ_110_ and γ_430_. Also in the second generation (S2) a significantly higher global methylation level compared to the control plants was present, but only after exposure to the highest dose rate. The significant increase in methylation percentage in the S1 also resulted in significant increases between the S1 and the other two generations at these same dose rates. In addition, a deeper analysis of the samples using WGBS was performed and the global methylation level was calculated using the WGBS result (Supplementary Figure S1). In general, these data followed the UPLC-MS/MS data but showed a higher variation and thus no significant difference were found within the WGBS global methylation levels. The global weighted methylation levels, calculated using methods described in Schultz et al. (2012), are shown in the Supplementary Table S1. The global weighted methylation levels in this study did not significantly vary between treatments. For control values these are on average ∼30% CG, ∼13.5% CHG, and ∼6.8% CHH, which is in line with previous studies in which DNA methylation levels in *A. thaliana* leaves is studied (Niederhuth et al., 2016; Bartels et al., 2018; Zhou M. et al., 2019).

### Differentially Methylated Regions Are Predominantly Found in CG Methylation Context and at Lowest Dose Rate and Later Generations

For the WGBS analyses a limited set of three dose rates were used (γ_0_ = control, γ_30_ = 30 mGy/h, and γ_110_ = 110 mGy/h). To check for global differences between generations or treatments, a pairwise comparison was performed looking for differences only between two different radiation treatments of the same generation (intragenerational) or two different generations for the same treatment (intergenerational). A distinction was made between the different methylation contexts for which the methylated cytosine (C) was either followed by a guanine (CG) or another nucleotide by a guanine (CHG) or not followed by a guanine (CHH).

In Tables 1, 2, the number of differential hyper- and hypomethylated regions (DMRs), respectively, is represented accounting for a methylation difference of >20%. This cut-off was chosen in order to ensure a definite methylation difference was being studied. A more extensive overview of DMRs with different cut-offs of methylation differences (5, 10, and 30%) can be found in the Supplementary Tables S2–S5. Highest differences were found for CG methylation, whereas only limited changes in CHG methylation occurred and no changes in CHH methylation were observed (Tables 1, 2). Furthermore, within the generations over the different dose rates (intragenerational), the largest number of DMR changes occurred between the γ_0_ and γ_30_ groups, with the majority occurring in S2 generation (i.e., 473 hypermethylated DMRs, 316 hypomethylated DMRs in S2γ_0_ compared to S2γ_30_). However, it is important to note that no significant changes are observed in the parent generation after exposure to IR. Intergenerationally, the largest number of (both hypo- as hypermethylated) DMRs occurred between generations after exposure to γ_30_, with the major change between S1γ_30_ vs. S2γ_30_ (1,057 hypermethylated DMRs in the second generation compared to the first generation, whereas 833 DMRs were found to be hypomethylated). Here, it is also clear that no changes occur in the γ_0_ group and few changes occur in the γ_110_ group.

**TABLE 1:** The number of hypermethylated DMRs (sorted by methylation context) that were identified after the comparison of the entire methylome of *A. thaliana*.

Hypermethylated							
Intragenerational (dose rate effects)				Intergenerational (generation effects)			

	CG	CHG	CHH		CG	CHG	CHH
P0γ_0_ vs. P0γ_30_	0	0	0	P0γ_0_ vs. S1γ_0_	0	0	0
P0γ_0_ vs. P0γ_110_	0	0	0	P0γ_0_ vs. S2γ_0_	0	0	0
P0γ_30_ vs. P0γ_110_	0	0	0	S1γ_0_ vs. S2γ_0_	0	0	0
S1γ_0_ vs. S1γ_30_	69	0	0	P0γ_30_ vs. S1γ_30_	92	1	0
S1γ_0_ vs. S1γ_110_	2	0	0	P0γ_30_ vs. S2γ_30_	176	1	0
S1γ_30_ vs. S1γ_110_	21	0	0	S1γ_30_ vs. S2γ_30_	1,057	2	0
S2γ_0_ vs. S2γ_30_	473	1	0	P0γ_110_ vs. S1γ_110_	0	0	0
S2γ_0_ vs. S2γ_110_	1	0	0	P0γ_110_ vs. S2γ_110_	0	0	0
S2γ_30_ vs. S2γ_110_	7	0	0	S1γ_110_ vs. S2γ_110_	6	0	0

*Eighteen pairwise comparisons were made either between generations [P0 (Parent generation), S1 (generation 1), S2 (generation 2)] for the same dose rate {γ_30_ (30 mGy/h), γ_110_ (110 mGy/h), γ_0_ [control condition (<0.1 μGy/h)] or between different dose rates of the same generation (Methylation difference of > 20%) (p ≤ 0.05)}.*

**TABLE 2:** The number of hypomethylated DMRs (sorted by methylation context) that were identified after the comparison of the entire methylome of *A. thaliana*.

Hypomethylated							
Intragenerational (dose rate effects)				Intergenerational (generation effects)			

	CG	CHG	CHH		CG	CHG	CHH
P0γ_0_ vs. P0γ_30_	0	0	0	P0γ_0_ vs. S1γ_0_	0	0	0
P0γ_0_ vs. P0γ_110_	0	0	0	P0γ_0_ vs. S2γ_0_	0	0	0
P0γ_30_ vs. P0γ_110_	0	0	0	S1γ_0_ vs. S2γ_0_	0	0	0
S1γ_0_ vs. S1γ_30_	64	0	0	P0γ_30_ vs. S1γ_30_	95	1	0
S1γ_0_ vs. S1γ_110_	0	0	0	P0γ_30_ vs. S2γ_30_	103	0	0
S1γ_30_ vs. S1γ_110_	17	0	0	S1γ_30_ vs. S2γ_30_	833	2	0
S2γ_0_ vs. S2γ_30_	316	0	0	P0γ_110_ vs. S1γ_110_	0	0	0
S2γ_0_ vs. S2γ_110_	5	0	0	P0γ_110_ vs. S2γ_110_	0	0	0
S2γ_30_ vs. S2γ_110_	8	0	0	S1γ_110_ vs. S2γ_110_	8	0	0

*Eighteen pairwise comparisons were made either between generations [P0 (Parent generation), S1 (generation 1), S2 (generation 2)] for the same dose rate {γ_30_ (30 mGy/h), γ_110_ (110 mGy/h), γ_0_ [control condition (<0.1 μGy/h)] or between different dose rates of the same generation (Methylation difference of > 20%) (p ≤ 0.05)}.*

### Differentially Methylated Regions Associated With Genes and Transposable Elements

For detailed analysis, DMRs were split up into those associated with the promoter associated region (<2 kbp upstream of the gene start), gene body, or TEs. Looking into the DMRs associated with the promoter region (Table 3), the highest number of affected genes (either hypo- of hypermethylated) are found over the γ_30_-exposed generations and more specifically in the second generation. Also between the control and γ_30_ dose rates, we see a higher number of DMRs associated with promoter regions. A similar observation was made in DMRs associated with the gene body i.e., DMRs that overlap, at least partially, with the gene body sequence (Table 3), with a strong generation effect resulting in 345 hypo- and 464 hypermethylated DMRs between S1γ_30_ and S2γ_30_. Additionally, the biggest dose-rate dependent effect was observed in S2 with 140 and 189 hypo- and hypermethylated DMRs in S2γ_0_ vs. S2γ_30_. Again, the highest dose rate (γ_110_) does not affect DMRs as strongly as γ_30_. A list of genes associated with DMRs in their promoter regions and/or gene bodies can be accessed through Gene Expression Omnibus (GEO), as specified in the “Data Availability Statement” section.

**TABLE 3:** Number of genes with differentially methylated regions (DMRs) (CG) found in the promoter associated regions (<2 kbp upstream), gene bodies, and transposable elements (TEs) of *A. thaliana* divided in hypo- and hyper methylation.

Intergenerational (generational effect)						
	Hypo			Hyper		
Comparison	Promoter associated region	Gene body	TEs	Promoter associated region	Gene body	TEs
P0γ_30_ vs. S1γ_30_	33	34	15	31	38	12
P0γ_30_ vs. S2γ_30_	34	42	5	69	60	35
S1γ_30_ vs. S2γ_30_	255	345	95	327	464	134
S1γ_110_ vs. S2γ_110_	0	3	0	5	1	3
**Intragenerational (dose rate effects)**						
S1γ_0_ vs. S1γ_30_	31	20	10	27	26	14
S1γ_0_ vs. S1γ_110_	0	0	0	1	0	0
S1γ_30_ vs. S1γ_110_	7	6	4	7	12	4
S2γ_0_ vs. S2γ_30_	90	140	23	165	189	75
S2γ_0_ vs. S2γ_110_	3	2	2	1	0	0
S2γ_30_ vs. S2γ_110_	2	2	1	1	3	0

*Methylation difference of > 20%) (p ≤ 0.05) {γ_30_ (30 mGy/h), γ_110_ (110 mGy/h), γ_0_ [control condition (<0.1 μGy/h)], P0 (Parent generation), S1 (generation 1), S2 (generation 2).*

The link between DMRs and TEs was studied by selecting for DMRs located within or at least overlapping with TE sequences. From this data it was observed that a large number of DMRs were associated with TEs, with the majority of them being hyper methylated (Table 3).

**FIGURE 3:**
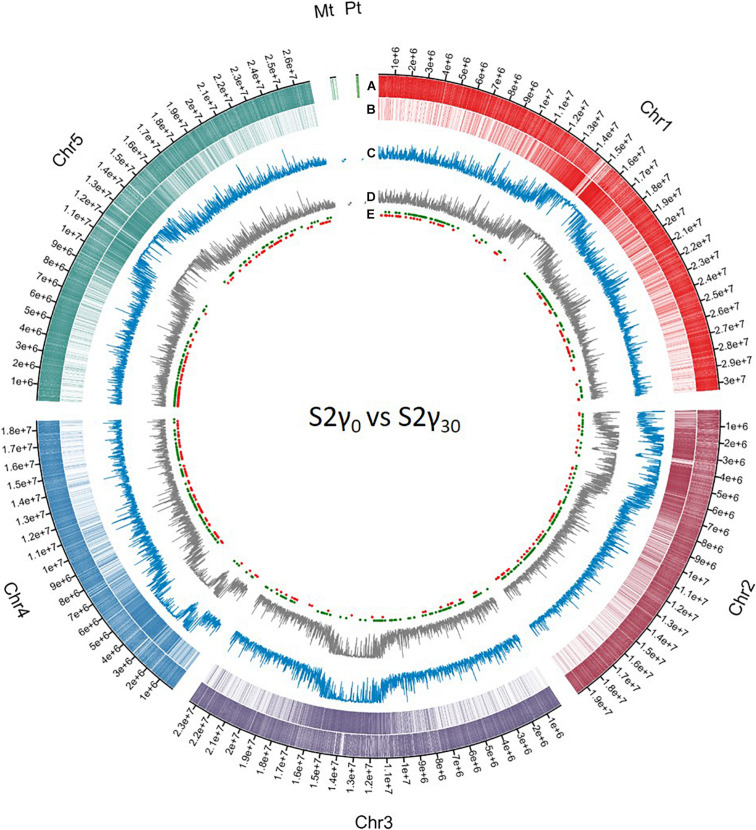
Circos representation of DNA methylation locations on the different chromosomes and the mitochondrial genome (Mt) and the plastid (Pt) coming from whole genome bisulfite sequencing data. The first **(A)** and second layer **(B)** represent the genes and transposable elements per chromosome, respectively, differently coloured per chromosome. The third **(C)** and fourth **(D)** layer display the methylation level averaged over a window of 10,000 bp for S2γ_30_ and S2γ_0_, respectively, y-axis from 0 to 1. The fifth layer **(E)** shows the different differentially methylated regions (DMRs) as identified in the current analysis, hypermethylated and hypomethylated DMRs are represented by green dots and red dots, respectively. Circos plot was created using Circa software (http://omgenomics.com/circa).

Similar as to what is observed for genes associated with DMRs (either in the promoter region or the gene body), the second generation has the highest number of TEs associated with DMRs. In addition, the plants in the γ_30_ condition have a higher number of differentially methylated TEs compared to those in the control and γ_110_ groups. However, in the case of the TEs, there is a stronger link with hypermethylation than hypomethylation than was seen in the genes. A list of the affected TEs can be accessed through Gene Expression Omnibus (GEO), as specified in the “Data Availability Statement” section. The global methylation level including the location of the DMRs per chromosome for S2γ_0_ vs. S2γ_30_, as identified in this analysis, is represented in Figure 3, similar representations for the comparisons P0γ_30_ vs. S1γ_30_, P0γ_30_ vs. S2γ_30_, and S1γ_30_ vs. S2γ_30_ can be found in the Supplementary Figures S2–S4. The average methylation levels over the different regions of the genome (<2 kb upstream promoter-associated region, gene body, and the region 2 kb downstream from the gene) per condition and per methylation context are presented in the Supplementary Figure S5. As expected, CG methylation is the biggest contributor in the gene methylation.

### Genes With Differentially Methylated Regions Linked to Stress Responses

The gene ontology (GO) term enrichment of genes associated with DMRs was split between those with affected promoter regions and those with affected gene bodies. Each was also divided into hypo- and hypermethylated DMRs. The location of DNA methylation in respect to a gene is important to its regulatory function, as DMRs located in the promoter-associated region will have a different effect than those found in the gene body. By looking at these DMRs individually based on their location, it will give a better insight into the biological processes that are affected after exposure to IR in specific generations (intragenerational, different dose rates) or over three generations (intergenerational, within one dose rate).

**FIGURE 4:**
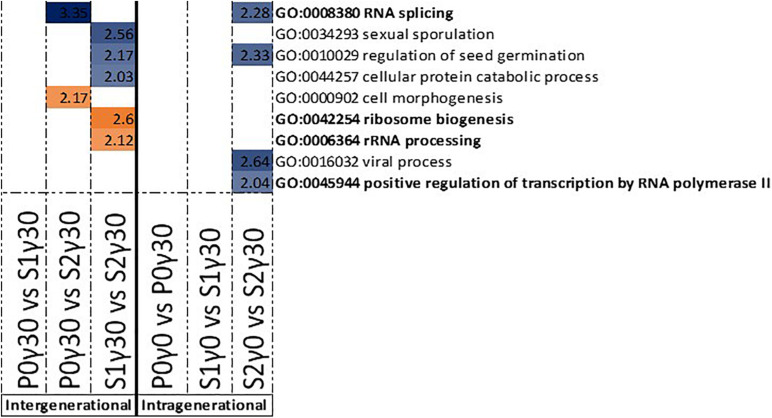
Gene ontology (GO) term enrichment for hypo- and hypermethylated DMRs in the promoter-associated regions of *Arabidopsis thaliana* for the intergenerational comparisons P0γ_30_ vs. S1γ_30_, P0γ_30_ vs. S2γ_30_, and S1γ_30_ vs. S2γ_30_ (on the left), and the intragenerational comparisons P0γ_0_ vs. P0γ_30_, S1γ_0_ vs. S1γ_30_, S2γ_0_ vs. S2γ_30_ (on the right) {γ_30_ (30 mGy/h), γ_0_ [control condition (<0.1 μGy/h)], P0 (Parent generation), S1 (generation 1), S2 (generation 2)}. S1 came from a previously exposed generation and S2 came from a line with two previously exposed generations. The –log_1__0_(P) value is shown and shaded according to its value per GO term, blue represents hypermethylation, whereas orange represents hypomethylation. GO terms highlighted in bold are those discussed in this paper.

In the intergenerational GO enrichment analysis for promotor regions (Figure 4), an enrichment for “ribosome biogenesis” and “rRNA processing” was observed in the hypomethylated DMRs in promotor regions between S1γ_30_ vs. S2γ_30_. For the hypermethylated DMRs in the promoter regions, an enrichment for “RNA splicing” is observed in P0γ_30_ vs. S2γ_30_ (Figure 4). An enrichment for “RNA splicing” is also observed in the intragenerational analysis (S2γ_0_ vs. S2γ_30_) of hypermethylated DMRs in the promoter regions along with an enrichment for the “positive regulation of transcription by RNA polymerase II” (Figure 4). The intragenerational analysis of hypomethylated DMRs in the promoter-associated regions yielded no significantly enriched GO terms.

**FIGURE 5:**
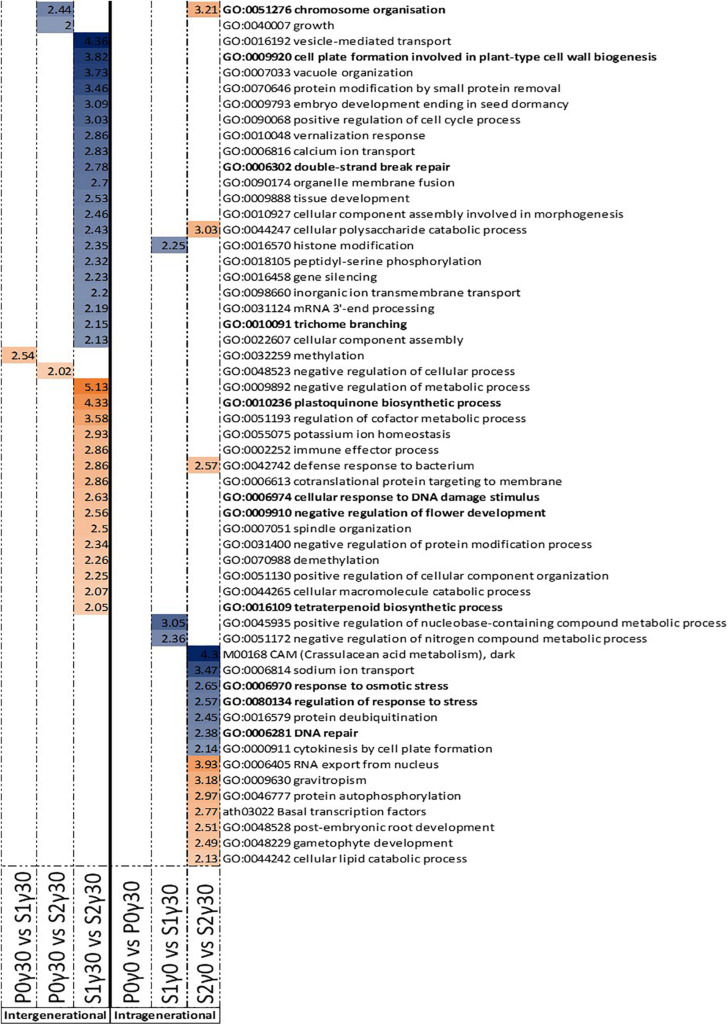
Gene ontology (GO) term enrichment for hypo- and hypermethylated DMRs in the gene bodies of *Arabidopsis thaliana* for the intergenerational comparisons P0γ_30_ vs. S1γ_30_, P0γ_30_ vs. S2γ_30_, and S1γ_30_ vs. S2γ_30_ (on the left), and the intragenerational comparisons P0γ_0_ vs. P0γ_30_, S1γ_0_ vs. S1γ_30_, S2γ_0_ vs. S2γ_30_ (on the right) {γ_30_ (30 mGy/h), γ_0_ [control condition (<0.1 μGy/h)], P0 (Parent generation), S1 (generation 1), S2 (generation 2)}. S1 came from a previously exposed generation and S2 came from a line with two previously exposed generations. The –log_1__0_(P) value is shown and shaded according to its value per GO term, blue represents hypermethylation, whereas orange represents hypomethylation. GO terms highlighted in bold are those discussed in this paper.

For the DMRs in gene bodies, the intergenerational GO term analysis of the hypermethylated DMRs shows an enrichment for “chromosome organisation” in the comparison between the second generation and previously unexposed plants of the parent generation (P0γ_30_ vs. S2γ_30_) (Figure 5). Between the second generation and first generation (S1γ_30_ vs. S2γ_30_), an enrichment for “cell plate formation in plant-type cell wall biogenesis,” “double-strand break repair,” and “trichrome branching” is observed in the hypermethylated DMRs (Figure 5). For the hypomethylated DMRs of the gene bodies an enrichment for “plastoquinone biosynthetic process,” “cellular response to DNA damage stimulus,” “negative regulation of flower development,” and “tetraterpenoid biosynthesis” is observed, in the comparison between the first and second generation of γ_30_-exposed plants (S1γ_30_ vs. S2γ_30_) (Figure 5).

The intragenerational GO enrichment analysis of the hypermethylated DMRs associated with gene bodies shows an enrichment for the “response to osmotic stress,” “regulation of response to stress,” and “DNA repair” in the comparison within the second generation between the control plants and γ_30_-exposed plants (S2γ_0_ vs. S2γ_30_) (Figure 5). In the hypomethylated DMRs in the gene bodies an enrichment for “chromosome organisation” is observed in the second generation between the control and γ_30_ group (S2γ_0_ vs. S2γ_30_) (Figure 5).

In the Supplementary Figures S6, S8 Venn diagrams of overlapping genes (for both those affected by promoter associated DMRs and those with gene body associated DMRs) between the different comparisons within γ_30_ (P0γ_30_ vs. S2γ_30_, P0γ_30_ vs. S1γ_30_, S1γ_30_ vs. S2γ_30_) can be found. Additionally, the GO enrichment can be found of those overlapping genes (Supplementary Figures S7, S9). Only a small overlap is observed over the three generations for differentially methylated promoter regions (Supplementary Figure S6 and Supplementary Tables S6, S7), except for those in P0γ_30_ vs. S2γ_30_ and S1γ_30_ vs. S2γ_30_. Here, 19 overlapping genes with hypomethylated DMRs in their promoter regions and 18 with hypermethylated DMRs in their promoter regions are found (Supplementary Figures S6A,B). This overlap of genes with hypermethylated DMRs in their promoter regions shows an enrichment for “RNA splicing,” thereby showing that there is an involvement of IR-induced DNA methylation in the regulation of this process (Supplementary Figure S7). The hypomethylated ones are enriched for “cell differentiation,” a regular day-to-day process (Supplementary Figure S7). The study into any overlap in genes with DMRs in their gene bodies over the three generations in the γ_30_ condition showed a similar result with most of the overlap occurring between P0γ_30_ vs. S2γ_30_ and S1γ_30_ vs. S2γ_30_ (28 hypomethylated and 47 hypermethylated) (Supplementary Figures S8A,B). The GO enrichment study for these genes shows an enrichment for “response to osmotic stress” and “DNA repair” (Supplementary Figure S9).

## Discussion

It was hypothesised that the exposure to IR would induce DNA methylation changes in plants and that these DNA methylation profiles differ between generations with a different IR exposure history. Based on the UPLC-MS/MS analysis a significantly increased global methylation level is observed mainly in the first generation (S1) plants, which were exposed to IR compared to the parent generation but decreased again in the following generation (S2) (Figure 2). Additionally, the parent generation and second generation showed no significant differences compared to each other, hereby indicating that when considering global methylation levels changes in methylation percentage predominantly happen in the exposed first generation (S1). Such a strong response in S1 would fit with findings of a previous study, where oxidative stress and cell wall-related enzyme activity was also increased in the exposed first generation of *A. thaliana* plants (van de Walle et al., 2016). A study done on *Daphnia magna* exposed to chronic gamma radiation, also showed an increase in the number of DNA methylation changes in the first generations which tapered off in later generations (Trijau et al., 2018).

By analysing the number of DMRs as identified after WGBS (Tables 1, 2), most DMRs were found in both IR-exposed S1 and S2 and no DMRs were observed in the non-exposed plants over the different generations. As no stress-induced responses are expected between control groups, these findings validate the control group. However, also no DMRs are identified in the parent generation after exposure to IR which is comparable to the results on global methylation (UPLC-MS/MS). Taken together both the data of the UPLC-MS/MS and those obtained after WGBS thus indicate that, at least within the current set-up, there is a need for an initial exposure that acts as a form of priming in which a first exposure will only elicit a response in the following generation or exposure. A similar initial priming was observed in previous studies for plants in response toward predators as well as other (a)biotic stresses (van Hulten et al., 2006; Rasmann et al., 2012; Lamke and Baurle, 2017; Thomas and Puthur, 2017; Baurle, 2018). The fact that the second generation (S2) generally shows a markedly higher number of DMRs than the other two generations (Tables 1, 2) after exposure to IR, indicates the presence of a generation-dependent dose-rate effect. This could point towards an ongoing adaptive response, which will likely reach an equilibrium over a number of generations. The strong response found in the WGBS data in S2 is potentially not picked up in the UPLC-MS/MS data which only gives a global DNA methylation percentage in which local hypo- and/or hypermethylation changes will cancel each other out, and therefore are not taken into account.

For the DMRs of the S1 and S2 generation, no clear dose-rate dependent response was found (Tables 1, 2) in contrast the strongest effects were observed in the lower dose rate (γ_30_) compared to the control condition (γ_0_) and very little DMRs were present in the comparisons with γ_110_ (Tables 1, 2). Nonetheless, the plants in the γ_110_ group did show a normal growth (and biomass) similar to the other plants, as was also seen under the same conditions and set-up by van de Walle et al. (2016). This lack of DMRs in the highest dose rate (γ_110_) could indicate that a certain threshold is crossed at which the plants switch to a different method of coping with the IR exposure. Kumagai et al. (2000) found some potential indications of the existence of such a threshold when studying seed germination of *A. thaliana* plants irradiated at different dose rates. They saw a gradual decrease in germination rate with increasing dose rate (2–9 kGy), however, at a certain dose rate (10 kGy) the germination rate suddenly dropped to zero. Comparably, IR-exposed *Lemna minor* plants shifted from acclimation to a survival strategy by expressing higher levels of antioxidant defence and DNA repair genes, at the higher dose rates (>232 mGy/h) (Van Hoeck et al., 2017). A transient response has also been observed in response to other stress conditions, such as salinity and UV-B irradiation (Munns and Gilliham, 2015; Mosadegh et al., 2019). Based on the current experimental design it is hypothesised that DNA methylation plays a more prominent role in the regulation of the plant response to lower dose rates than the higher ones. This hypothesis however, needs further testing for more doses and/or time points or confirming it in other plant species.

From the intra- and intergenerational comparison across different gamma exposure conditions, it is clear that most changes occur in the CG methylation context (Tables 1, 2). Only a limited number of CHG DMRs are present and no changes in CHH methylation were observed. The fact that IR seems to only affect CG methylation is an interesting discovery. Research has shown that in plants the environmental stress conditions can affect each methylation context differently (Niederhuth and Schmitz, 2017; Bartels et al., 2018). A study done on pathogen stress in *A. thaliana*, for instance, showed that upon infection CG and CHG levels were similar to the control group, whereas CHH methylation varied more among the samples, thereby showing CHH methylation to be more responsive to this biotic stress inducing agent (Dowen et al., 2012). Other studies showed differential DNA methylation contexts as a result of abiotic stress, e.g., differential DNA methylation in the CHH context as a result of cold stress in *Antirrhinum majus* (Hashida et al., 2006), or drought stress in *Solanum lycopersicum* (González et al., 2011), or in the CHG context as a result of salinity stress in *Mesembryanthenum crystallinum* (Dyachenko et al., 2006). This indicates that more research is needed to clarify the specific role of the cytosine methylation context in the response to stress. However, CG methylation has been shown to be very stable compared to the other methylation contexts and inheritance of CG methylation has been observed to play a key role in transferring epigenetic information to the following generations (Saze et al., 2003; Vaillant and Paszkowski, 2007). Hence, as mainly CG methylation was observed here, a potential inheritable epigenetic IR-stress response is occurring. The exact molecular mechanism behind this preference for CG methylation is yet to be studied, however, the METHYLTRANSFERASE1 (MET1) might play a role in this. MET1 is the CG methylation maintenance methyltransferase in *A. thaliana* and is also involved in *de novo* DNA methylation (Finnegan and Kovac, 2000; Gehring and Henikoff, 2008). The link with IR-stress response has already been made in a previous study where they saw an upregulation of MET1, as well as CMT3 (CHROMOMETHYLASE 3) and SUVH5 [SU(VAR)3-9 HOMOLOG 5] in *A. thaliana* plants exposed to IR (Sidler et al., 2015). In addition, there is a possibility that DNA glycosylase/AP lyase ROS1 plays a role in the active demethylation of different methylation contexts (Kim et al., 2019). However, as ROS1 does not only target CG methylation but also CHG and CHH contexts, be it at lower rates, it cannot be solely responsible for this CG methylation preference (Gong et al., 2002; Agius et al., 2006; Morales-Ruiz et al., 2006; Tang et al., 2016; Kim et al., 2019).

In general more hypermethylated than hypomethylated DMRs were observed in the current study. This corresponds with earlier reports where the offspring of stressed plants showed hypermethylation under salt stress, pathogens, and IR stress (Kovalchuk et al., 2003; Boyko et al., 2007; Bilichak et al., 2012; Volkova et al., 2018) and is consistent with the higher global methylation level as determined by UPLC-MS/MS. Zooming in on specific DNA regions, the ratio of hyper- vs. hypomethylated DMRs can, however, vary. For example, a substantial number of DMRs associated with TEs were found in the intergenerational comparisons after exposure to γ_30_ (30 mGy/h) and in the intragenerational comparison in the second generation (S2) between the control and γ_30_ conditions, with the majority of them being hypermethylated (Table 3). This hypermethylation will likely lead to transcriptional silencing and therefore limiting expression and mobilisation of TEs, resulting in less genomic reshuffling (Sigman and Slotkin, 2016). A hypermethylation in response to IR exposure, has been previously hypothesised to act as a protective measure to increase genome stability (Kovalchuk et al., 2004; Boyko et al., 2007; Horemans et al., 2018; Volkova et al., 2018).

As the comparisons with the highest dose rate (γ_110_) yielded no GO term enrichments and as most significantly affected and stress related GO terms were found for the S2 generation, the focus of the following part of the discussion will lie on the comparison between the control (γ_0_) and γ_30_ conditions and mostly on the second generation and γ_30_ condition, unless stated otherwise. A significant number of enriched GO terms were found that could all be linked to RNA splicing and DNA repair. It is to our knowledge the first time that a DNA methylation driven regulation of both RNA splicing and DNA damage repair mechanisms is reported in plants exposed to IR over multiple generations. Alternative RNA splicing is often used in regulating stress-related genes in order to adjust to the stressor, thereby giving the plant a dynamic tool to respond to changing environmental situations (Staiger, 2015; Calixto et al., 2018; Laloum et al., 2018; Huertas et al., 2019). Combining ribosome biogenesis, rRNA processing (P0γ_30_ vs. S2γ_30_), and positive regulation of transcription of RNA polymerase II (S2γ_0_ vs. S2γ_30_) with the RNA splicing (P0γ_30_ vs. S2γ_30_ and S2γ_0_ vs. S2γ_30_), a potential stress (signalling) response is occurring over the three generations as well as intragenerationally between the control and γ_30_ condition. However, exactly how these mechanisms react to IR and if/how the hypo- or hypermethylation of the promoter regions affects them, needs to be studied in more detail.

A DNA repair response is regularly seen in IR-irradiated plants (Esnault et al., 2010; Gicquel et al., 2012; Dona et al., 2013; Georgieva et al., 2017). An enrichment for “DNA repair” was observed in the hypermethylated gene bodies of the second (S2) generation between the control group and the lowest dose rate. Additionally, “chromosome organisation” was found in both S2γ_0_ vs. S2γ_30_ hypomethylated gene bodies and P0γ_30_ vs. S2γ_30_ hypermethylated gene bodies The latter process has previously been found to be part of the plant IR-stress response (Shirley et al., 1992; Shikazono et al., 2001), and is involved in chromatin maintenance and modifications as well as DNA repair (Kim, 2019). Further, the hypomethylated gene bodies’ GO term enrichment between the first (S1) and second (S2) generation in the γ_30_ condition which contains the “cellular response to DNA damage stimulus” were found (Figure 5). Taken together these GO enrichments indicate DNA methylation is playing a regulating role in the DNA repair response. A few of the identified genes in our data (or homologues thereof) have been shown to be upregulated by IR in previous studies (e.g., *PARP-1*, *BRCA*) (Garcia et al., 2003; Culligan et al., 2006). In this study, a number of DNA repair and DNA damage response genes were tested (e.g., PARP1 and PARP2, data not shown). However, no direct correlation with DNA methylation levels were seen. Recently it was shown that gene associated DNA methylation resulted in a significantly delayed effect on actual gene expression (Atighi et al., 2020). In the current study we only harvested one sampling point per generation and therefore cannot corroborate this delayed effect on gene expression. Nonetheless, the fact that our data on differential DNA methylation do not directly link up with gene expression data from the harvest time point is in line with Atighi et al. (2020).

In addition to RNA splicing and DNA repair, a number of stress-related processes were found in the GO-enrichment analysis including “cell plate formation in plant-type cell wall biogenesis,” “trichome branching,” “plastoquinone biosynthetic process,” “tetraterpenoid biosynthesis,” and “negative regulation of flower development” (Figure 5). The fact that many DMRs are correlated with different genes and their process, including stress response, indicates that IR-induced DNA methylation is not random and indicates that regulation through changes in DNA methylation plays an important regulating role in the response of plants to IR, either by increasing genetic stability and/or regulating stress response gene expression.

Enrichment of GO-term “trichome branching” after exposure of multiple generations links IR-induced DNA methylation to the induction of trichome branching and is in agreement with a previous study that indicating the association of trichome density with epigenetic inheritance in plants (Scoville et al., 2011). Goh et al. (2014) showed that the number of trichomes increased dramatically in response to 200 Gy applied either chronically (1 week) or acutely (1 h). An enrichment between the S1 and S2 generation in the hypomethylated gene bodies was found for “plastoquinone biosynthetic process” and “tetraterpenoid biosynthesis” (Figure 5). The regulation of plastoquinone biosynthesis might protect plants from IR-damage to the photosynthetic apparatus which has been shown to be affected under IR (Gicquel et al., 2011; Vanhoudt et al., 2014). Induction of antioxidants and secondary metabolites including phenolic compounds, terpenoids and nitrogen-containing compounds have also been reported in this respect (Dixit et al., 2010; Popovic et al., 2013; Taheri et al., 2014; Vardhan and Shukla, 2017; Gudkov et al., 2019). In some organisms, including humans, carotenoids and lycopenes have shown a potential as radioprotectant (Islamian and Mehrali, 2015). The enrichment for terpenoid production can potentially also be linked to the aforementioned increased trichome accumulation as specific glandular trichomes have been shown to accumulate specific terpenoid molecules in response and adaptation to stress (Tang et al., 2020).

Intragenerationally, the “response to osmotic stress” was found in the second (S2) generation between the control and γ_30_ condition (Figure 5). Our findings correspond with the study of Rejili et al. (2008) that showed increased growth of IR-exposed *Medicago sativa* under high salinity. In addition, in *A. thaliana* plants irradiated with a gamma dose of 50 Gy, an improved tolerance to salinity, by regulating, amongst others, stress signal responses was reported (Qi et al., 2014). These studies indicate a form of priming to salinity stress by exposing the plants to IR. The more general “regulation of response to stress” includes a number of these above-mentioned osmotic stress response genes. Additionally, a significant number of the genes is associated with oxidative stress. This corresponds with the literature, in which an upregulation of certain oxidative stress response genes and antioxidant components in plants exposed to IR is observed (Van Hoeck et al., 2015; Einor et al., 2016; Volkova et al., 2017).

Lastly, an enrichment for the “negative regulation of flower development” is observed in the hypomethylated gene bodies between the first (S1) and second (S2) generation (Figure 5). The flowering response to IR is still under debate as studies have shown either an earlier or a later floral induction (Sax, 1955; Gunckel, 1957; Daly and Thompson, 1975; Kovalchuk et al., 2007; Hwang et al., 2016; Kryvokhyzha et al., 2018). The timing and regulation of flowering is important as it will affect the survivability of the next generation. Earlier flowering leads to quicker seed production and therefore secures the next generation. In some cases, seeds have been shown to be more stress resistant, however, under IR this is still controversial (Maity et al., 2009; Melki and Marouani, 2009; Moussa, 2011; Pozolotina et al., 2012). Alongside, premature flowering can also result in a reduced number and/or mass of the seeds (Huijser and Schmid, 2011). These studies’ findings therefore add to the existing literature on flowering under IR stress and indicate for the first time a potential role of IR-induced DNA methylation in the regulation of this process.

## Conclusion

In conclusion, our data are consistent with a potential regulating role for DNA methylation in the response of plants to IR in *Arabidopsis* plants exposed over multiple generations. The observed difference in response between γ_30_ and γ_110_, however, also indicates that studies on the effects of low dose IR on plants, specifically chronic irradiation within and over generations, are needed for helping in environmental risk assessments. As a follow up we suggest a kinetic study to detect responses shifted in time as well as experiments in which the multigenerational set-up will be combined with a transgenerational one. By including irradiated generations stemming from non-irradiated parent generations and vice versa, the analysis would conclusively separate generational/inherited DNA methylation from IR-induced DNA methylation. Secondly, the molecular mechanism behind the DNA methylation and its preference for CG methylation as a result of IR stress should be studied, for instance, by including gene expression analysis of relevant methyltransferases.

## Data Availability Statement

The raw bisulfite sequencing data and the processed data sets generated in this study have been deposited in the Gene Expression Omnibus (GEO) under accession GSE157965.

## Author Contributions

ES, NH, AC, and JV conceptualised and designed the project. JV, MV, and RN performed the practical experimental work. SV and GJ performed the UPLC-MS/MS analysis. FV performed the bisulfite sequencing. MM performed the bioinformatics analysis. PL, ES, NH, and AC contributed to the data interpretation. PL did the data analysis and research on biological relevance, and drafted the manuscript. All authors contributed in the revision.

## Conflict of Interest

The authors declare that the research was conducted in the absence of any commercial or financial relationships that could be construed as a potential conflict of interest.

## Footnotes

1 https://digitalinsights.qiagen.com

2 www.arabidopsis.org

3 http://bioinformatics.psb.ugent.be/webtools/Venn/

## Abbreviations

DMR: Differentially Methylated Region
GO: Gene Ontology
IR: Ionising Radiation
PAR: Photosynthetically Active Radiation
TE: Transposable Element
WGBS: Whole Genome Bisulfite Sequencing.
