# Supplementary material for: Changes in DNA Methylation in Arabidopsis thaliana Plants Exposed Over Multiple Generations to Gamma Radiation
Source: Front Plant Sci. 2021 Mar 31;12:611783. doi: 10.3389/fpls.2021.611783 (PMC8044457; doi:10.3389/fpls.2021.611783)
Supplement: Supplementary file 1 [file Data_Sheet_1.docx]

Supplementary Material

*The raw bisulphite sequencing data and the processed data sets generated in this study have been deposited in the Gene Expression Omnibus (GEO) under accession GSE157965.*


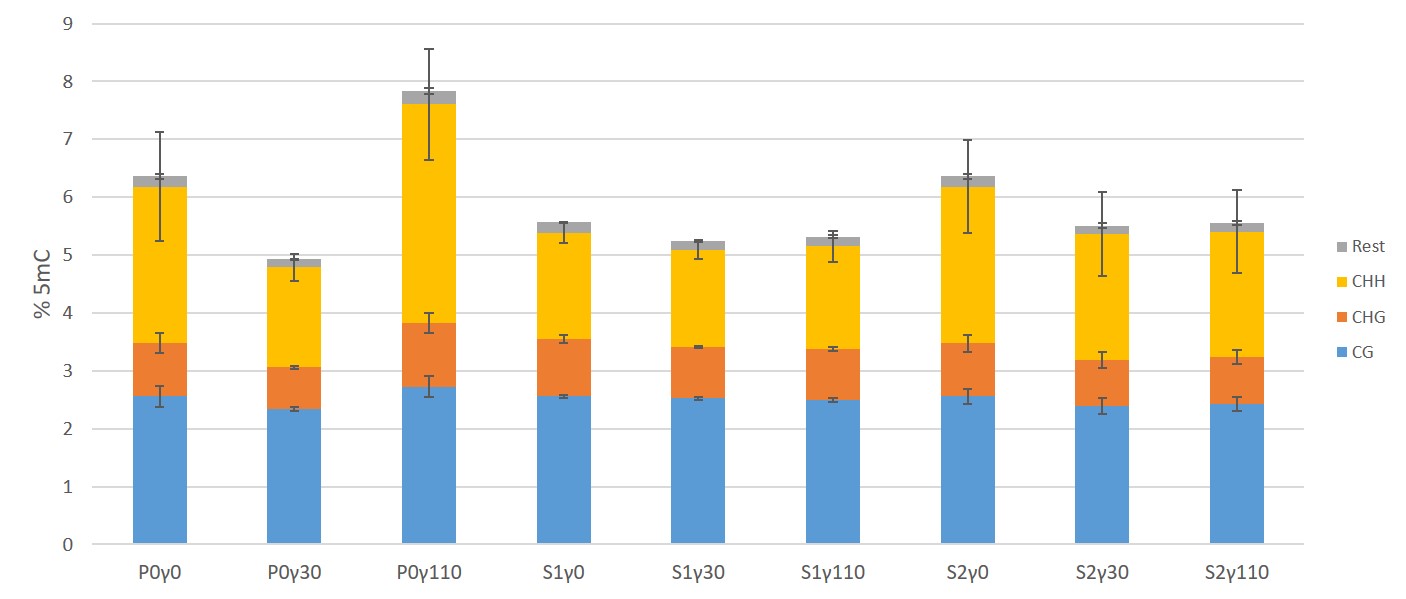


**Supplementary Figure S1:** *The global methylation percentage, determined with WGBS, of all generations (P0 (Parent generation), S1 (generation 1), S2 (generation 2)) of A. thaliana plants (γ_0_ = control condition (<0.1µGy/h), γ_30_ = 30 mGy/h, γ_110_= 110 mGy/h, the dose rates γ_60_ = 60 mGy/h and γ_430_ = 430 mGy/h were not included in the WGBS). Subdivision is made per methylation context (blue: CG, orange: CHG, yellow: CHH) and rest (grey), which represents methylation in an unidentified context. Values are represented relative to P0γ_0_ (effective values P0γ_0_ = 6.36%, S1γ_0_ = 4.50%, S2γ_0_ = 6.62%). Measurements are an average ± SE of 5 biological replicates.*

**Supplementary Table S1*:***  *The global weighted methylation level percentage determined with whole genome bisulfite sequencing (WGBS) of all generations (P0 (Parent generation), S1 (generation 1), S2 (generation 2)) of A. thaliana plants (γ_0_ = control condition (<0.1µGy/h), γ_30_ = 30 mGy/h, γ_110_= 110 mGy/h, the dose rates γ_60_ = 60 mGy/h and γ_430_ = 430 mGy/h were not included in the WGBS) divided by chromosomes 1-5, mitochondrial chromosome (Mt), and plastid (Pt) per methylation context (CG, CHG, CHH). The standard error is included for each case and is an average of 5 biological replicates.*

**Supplementary Table S2:** *The number of hypermethylated differentially methylated regions (DMRs) for CpG (sorted by methylation difference, 5%, 10%, 30%) that were identified after the comparison of the entire methylome of A. thaliana. 18 pairwise comparisons were made either between generations (P0 (Parent generation), S1 (generation 1), S2 (generation 2)) for the same dose rate (*γ_30_ *(30 mGy/h),* γ_110_ *(110 mGy/h),* γ_0_ *(control condition (<0.1µGy/h)) or between dose rates of one generation. (Methylation difference of >20%) (p≤0.05))*

| **Hypermethylated** | | | | | | | |
| --- | --- | --- | --- | --- | --- | --- | --- |
| **Intragenerational (Dose rate effects)** | | | | **Intergenerational (Generation effects)** | | | |
|  | **5%** | **10%** | **30%** |  | **5%** | **10%** | **30%** |
| P0γ_0_ vs P0γ_30_ | 0 | 0 | 0 | P0γ_0_ vs S1γ_0_ | 1 | 0 | 0 |
| P0γ_0_ vs P0γ_110_ | 0 | 0 | 0 | P0γ_0_ vs S2γ_0_ | 0 | 0 | 0 |
| P0γ_30_ vs P0γ_110_ | 0 | 0 | 0 | S1γ_0_ vs S2γ_0_ | 3 | 0 | 0 |
|  |  |  |  |  |  |  |  |
| S1γ_0_ vs S1γ_30_ | 134 | 119 | 35 | P0γ_30_ vs S1γ_30_ | 185 | 167 | 42 |
| S1γ_0_ vs S1γ_110_ | 3 | 3 | 1 | P0γ_30_ vs S2γ_30_ | 244 | 234 | 110 |
| S1γ_30_ vs S1γ_110_ | 36 | 32 | 13 | S1γ_30_ vs S2γ_30_ | 1718 | 1627 | 532 |
|  |  |  |  |  |  |  |  |
| S2γ_0_ vs S2γ_30_ | 670 | 632 | 253 | P0γ_110_ vs S1γ_110_ | 0 | 0 | 0 |
| S2γ_0_ vs S2γ_110_ | 2 | 2 | 1 | P0γ_110_ vs S2γ_110_ | 0 | 0 | 0 |
| S2γ_30_ vs S2γ_110_ | 9 | 7 | 7 | S1γ_110_ vs S2γ_110_ | 12 | 8 | 5 |

**Supplementary Table S3:** *The number of hypomethylated differentially methylated regions (DMRs) for CpG (sorted by methylation difference, 5%, 10%, 30%) that were identified after the comparison of the entire methylome of A. thaliana. 18 pairwise comparisons were made either between generations (P0 (Parent generation), S1 (generation 1), S2 (generation 2)) for the same dose rate (*γ_30_ *(30 mGy/h),* γ_110_ *(110 mGy/h),* γ_0_ *(control condition (<0.1µGy/h)) or between dose rates of one generation. (Methylation difference of >20%) (p≤0.05))*

| **Hypomethylated** | | | | | | | |
| --- | --- | --- | --- | --- | --- | --- | --- |
| **Intragenerational (Dose rate effects)** | | | | **Intergenerational (Generation effects)** | | | |
|  | **5%** | **10%** | **30%** |  | **5%** | **10%** | **30%** |
| P0γ_0_ vs P0γ_30_ | 1 | 0 | 0 | P0γ_0_ vs S1γ_0_ | 1 | 0 | 0 |
| P0γ_0_ vs P0γ_110_ | 0 | 0 | 0 | P0γ_0_ vs S2γ_0_ | 0 | 0 | 0 |
| P0γ_30_ vs P0γ_110_ | 0 | 0 | 0 | S1γ_0_ vs S2γ_0_ | 2 | 1 | 0 |
|  |  |  |  |  |  |  |  |
| S1γ_0_ vs S1γ_30_ | 95 | 93 | 39 | P0γ_30_ vs S1γ_30_ | 175 | 157 | 53 |
| S1γ_0_ vs S1γ_110_ | 2 | 1 | 0 | P0γ_30_ vs S2γ_30_ | 153 | 150 | 65 |
| S1γ_30_ vs S1γ_110_ | 39 | 20 | 11 | S1γ_30_ vs S2γ_30_ | 1382 | 1327 | 407 |
|  |  |  |  |  |  |  |  |
| S2γ_0_ vs S2γ_30_ | 451 | 433 | 175 | P0γ_110_ vs S1γ_110_ | 0 | 0 | 0 |
| S2γ_0_ vs S2γ_110_ | 6 | 5 | 4 | P0γ_110_ vs S2γ_110_ | 0 | 0 | 0 |
| S2γ_30_ vs S2γ_110_ | 9 | 9 | 5 | S1γ_110_ vs S2γ_110_ | 12 | 12 | 5 |

| **Hypermethylated** | | | | | | | |
| --- | --- | --- | --- | --- | --- | --- | --- |
| **Intragenerational (Dose rate effects)** | | | | **Intergenerational (Generation effects)** | | | |
|  | **5%** | **10%** | **30%** |  | **5%** | **10%** | **30%** |
| P0γ_0_ vs P0γ_30_ | 0 | 0 | 0 | P0γ_0_ vs S1γ_0_ | 0 | 0 | 0 |
| P0γ_0_ vs P0γ_110_ | 0 | 0 | 0 | P0γ_0_ vs S2γ_0_ | 0 | 0 | 0 |
| P0γ_30_ vs P0γ_110_ | 0 | 0 | 0 | S1γ_0_ vs S2γ_0_ | 2 | 1 | 0 |
|  |  |  |  |  |  |  |  |
| S1γ_0_ vs S1γ_30_ | 1 | 1 | 0 | P0γ_30_ vs S1γ_30_ | 2 | 2 | 1 |
| S1γ_0_ vs S1γ_110_ | 0 | 0 | 0 | P0γ_30_ vs S2γ_30_ | 1 | 1 | 1 |
| S1γ_30_ vs S1γ_110_ | 0 | 0 | 0 | S1γ_30_ vs S2γ_30_ | 3 | 3 | 2 |
|  |  |  |  |  |  |  |  |
| S2γ_0_ vs S2γ_30_ | 1 | 1 | 0 | P0γ_110_ vs S1γ_110_ | 0 | 0 | 0 |
| S2γ_0_ vs S2γ_110_ | 0 | 0 | 0 | P0γ_110_ vs S2γ_110_ | 0 | 0 | 0 |
| S2γ_30_ vs S2γ_110_ | 0 | 0 | 0 | S1γ_110_ vs S2γ_110_ | 0 | 0 | 0 |

**Supplementary Table S4:** *The number of hypermethylated differentially methylated regions (DMRs) for CHG (sorted by methylation difference, 5%, 10%, 30%) that were identified after the comparison of the entire methylome of A. thaliana. 18 pairwise comparisons were made either between generations (P0 (Parent generation), S1 (generation 1), S2 (generation 2)) for the same dose rate (*γ_30_ *(30 mGy/h),* γ_110_ *(110 mGy/h),* γ_0_ *(control condition (<0.1µGy/h)) or between dose rates of one generation. ((Methylation difference of >20%) (p≤0.05))*

**Supplementary Table S5:** *The number of hypomethylated differentially methylated regions (DMRs) for CHG (sorted by methylation difference, 5%, 10%, 30%) that were identified after the comparison of the entire methylome of A. thaliana. 18 pairwise comparisons were made either between generations (P0 (Parent generation), S1 (generation 1), S2 (generation 2)) for the same dose rate (*γ_30_ *(30 mGy/h),* γ_110_ *(110 mGy/h),* γ_0_ *(control condition (<0.1µGy/h)) or between dose rates of one generation. (Methylation difference of >20%) (p≤0.05))*

| **Hypomethylated** | | | | | | | |
| --- | --- | --- | --- | --- | --- | --- | --- |
| **Intragenerational (Dose rate effects)** | | | | **Intergenerational (Generation effects)** | | | |
|  | **5%** | **10%** | **30%** |  | **5%** | **10%** | **30%** |
| P0γ_0_ vs P0γ_30_ | 0 | 0 | 0 | P0γ_0_ vs S1γ_0_ | 0 | 0 | 0 |
| P0γ_0_ vs P0γ_110_ | 0 | 0 | 0 | P0γ_0_ vs S2γ_0_ | 0 | 0 | 0 |
| P0γ_30_ vs P0γ_110_ | 0 | 0 | 0 | S1γ_0_ vs S2γ_0_ | 0 | 0 | 0 |
|  |  |  |  |  |  |  |  |
| S1γ_0_ vs S1γ_30_ | 0 | 0 | 0 | P0γ_30_ vs S1γ_30_ | 5 | 3 | 0 |
| S1γ_0_ vs S1γ_110_ | 0 | 0 | 0 | P0γ_30_ vs S2γ_30_ | 0 | 0 | 0 |
| S1γ_30_ vs S1γ_110_ | 0 | 0 | 0 | S1γ_30_ vs S2γ_30_ | 2 | 2 | 1 |
|  |  |  |  |  |  |  |  |
| S2γ_0_ vs S2γ_30_ | 1 | 1 | 1 | P0γ_110_ vs S1γ_110_ | 0 | 0 | 0 |
| S2γ_0_ vs S2γ_110_ | 0 | 0 | 0 | P0γ_110_ vs S2γ_110_ | 0 | 0 | 0 |
| S2γ_30_ vs S2γ_110_ | 0 | 0 | 0 | S1γ_110_ vs S2γ_110_ | 0 | 0 | 0 |

**
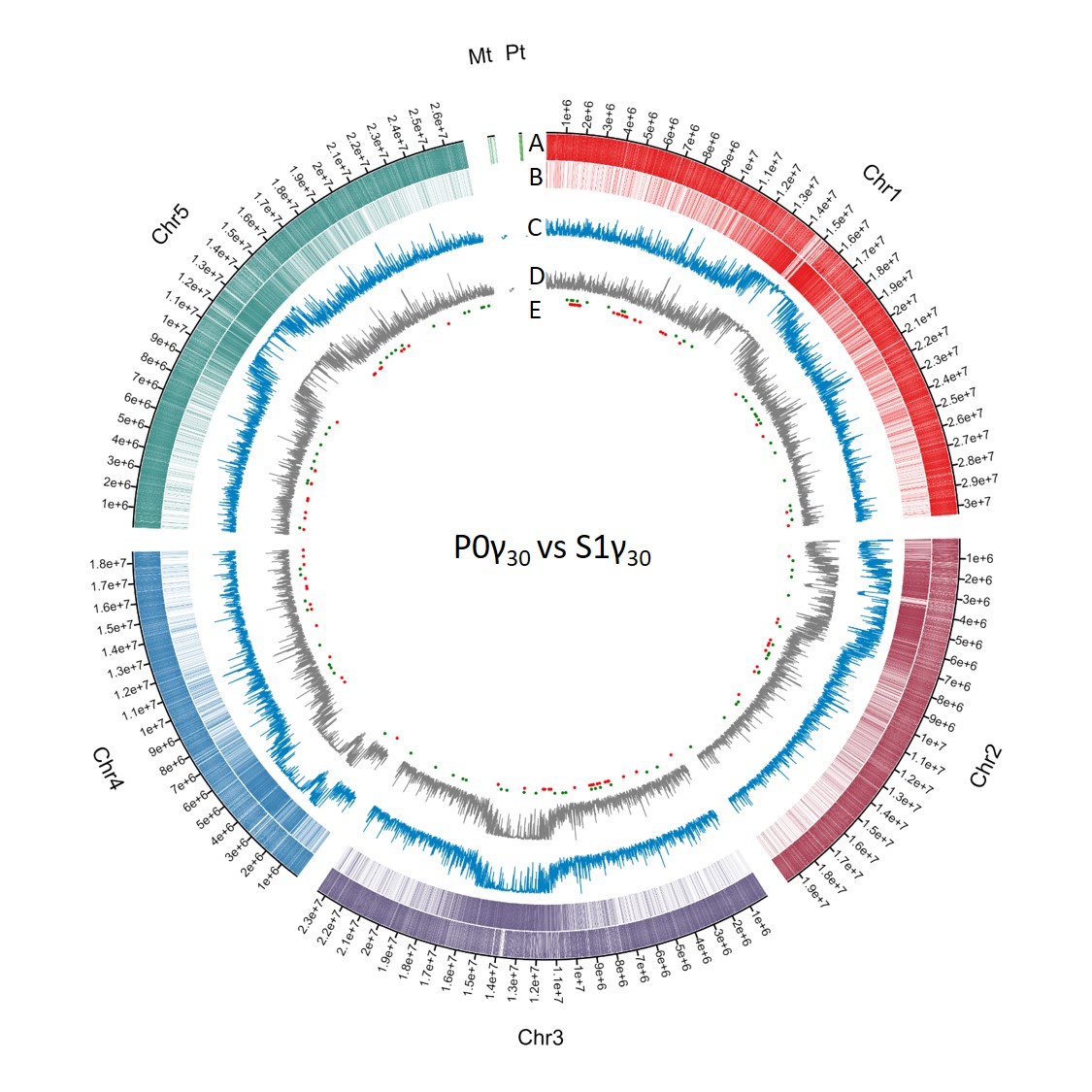
**

**Supplementary Figure S2:** *Circos representation of DNA methylation locations on the different chromosomes and the mitochondrial genome (Mt) and the plastid (Pt) coming from whole genome bisulfite sequencing data. The first (A) and second layer (B) represent the genes and transposable elements per chromosome, respectively, differently coloured per chromosome. The third (C) and fourth (D) layer display the methylation level averaged over a window of 10,000 bp for S1*γ_30_ *and P0*γ_30_, *respectively, y-axis from 0 to 1. The fifth layer (E) shows the different differentially methylated regions (DMRs) as identified in the current analysis, hypermethylated and hypomethylated DMRs are represented by green dots and red dots, respectively. Circos plot was created using Circa software (*[*http://omgenomics.com/circa*](http://omgenomics.com/circa)*).*

*
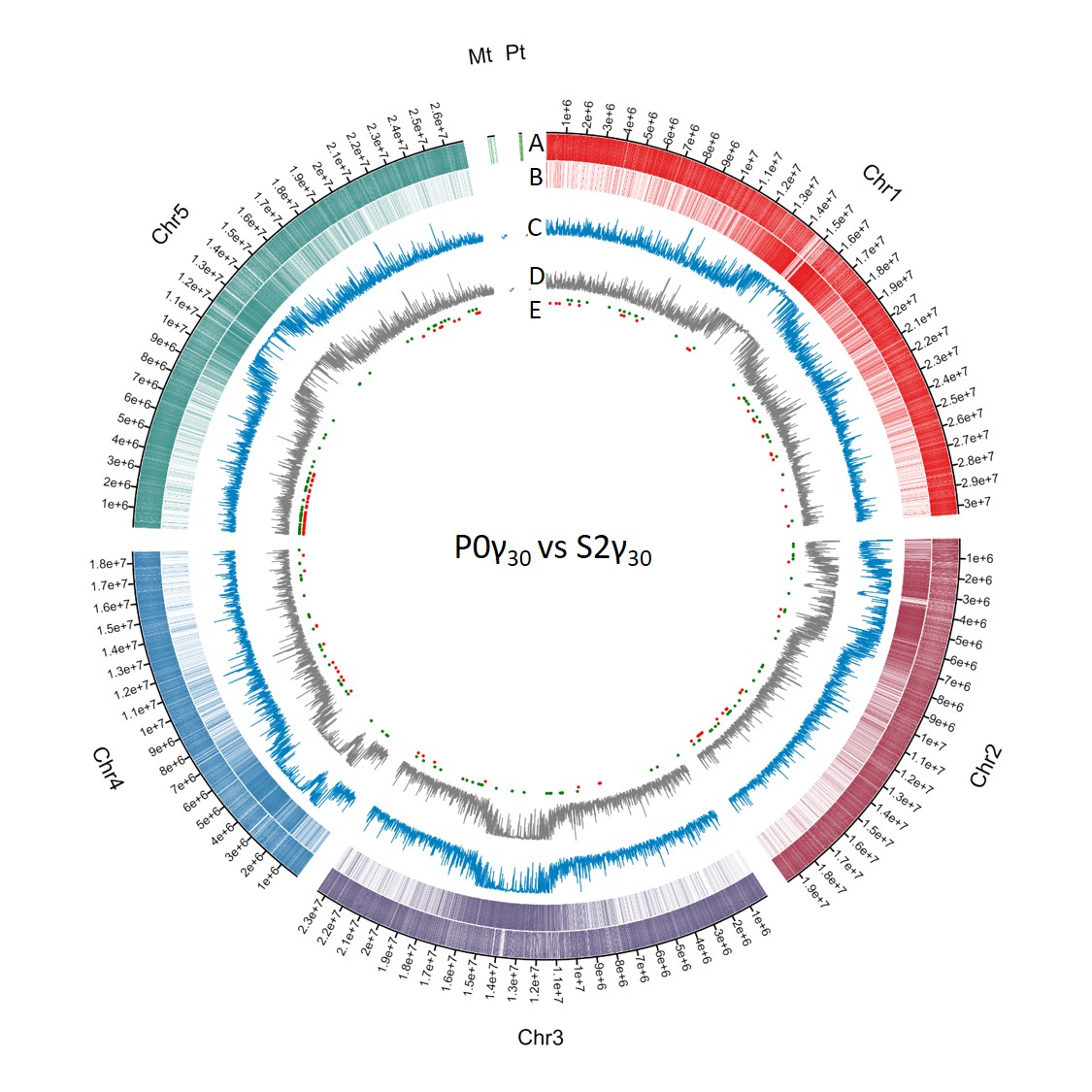
*

**Supplementary Figure S3:** *Circos representation of DNA methylation locations on the different chromosomes and the mitochondrial genome (Mt) and the plastid (Pt) coming from whole genome bisulfite sequencing data. The first (A) and second layer (B) represent the genes and transposable elements per chromosome, respectively, differently coloured per chromosome. The third (C) and fourth (D) layer display the methylation level averaged over a window of 10,000 bp for S2*γ_30_ *and P0*γ_30_, *respectively, y-axis from 0 to 1. The fifth layer (E) shows the different differentially methylated regions (DMRs) as identified in the current analysis, hypermethylated and hypomethylated DMRs are represented by green dots and red dots, respectively. Circos plot was created using Circa software (*[*http://omgenomics.com/circa*](http://omgenomics.com/circa)*).*

*
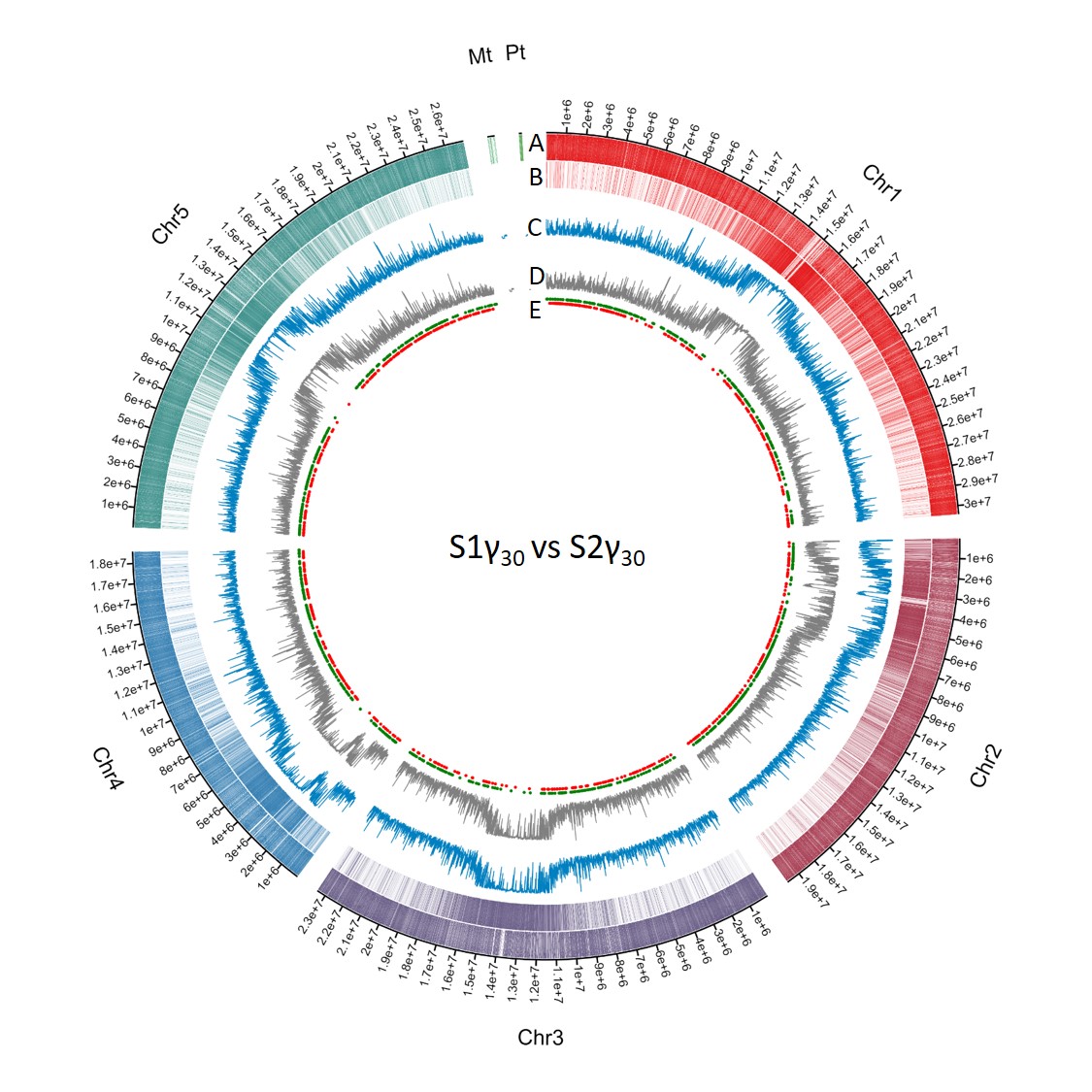
*

**Supplementary Figure S4:** *Circos representation of DNA methylation locations on the different chromosomes and the mitochondrial genome (Mt) and the plastid (Pt) coming from whole genome bisulfite sequencing data. The first (A) and second layer (B) represent the genes and transposable elements per chromosome, respectively, differently coloured per chromosome. The third (C) and fourth (D) layer display the methylation level averaged over a window of 10,000 bp for S2*γ_30_ *and S1*γ_30_, *respectively, y-axis from 0 to 1. The fifth layer (E) shows the different differentially methylated regions (DMRs) as identified in the current analysis, hypermethylated and hypomethylated DMRs are represented by green dots and red dots, respectively. Circos plot was created using Circa software (*[*http://omgenomics.com/circa*](http://omgenomics.com/circa)*).*

**Supplementary Figure S5:**
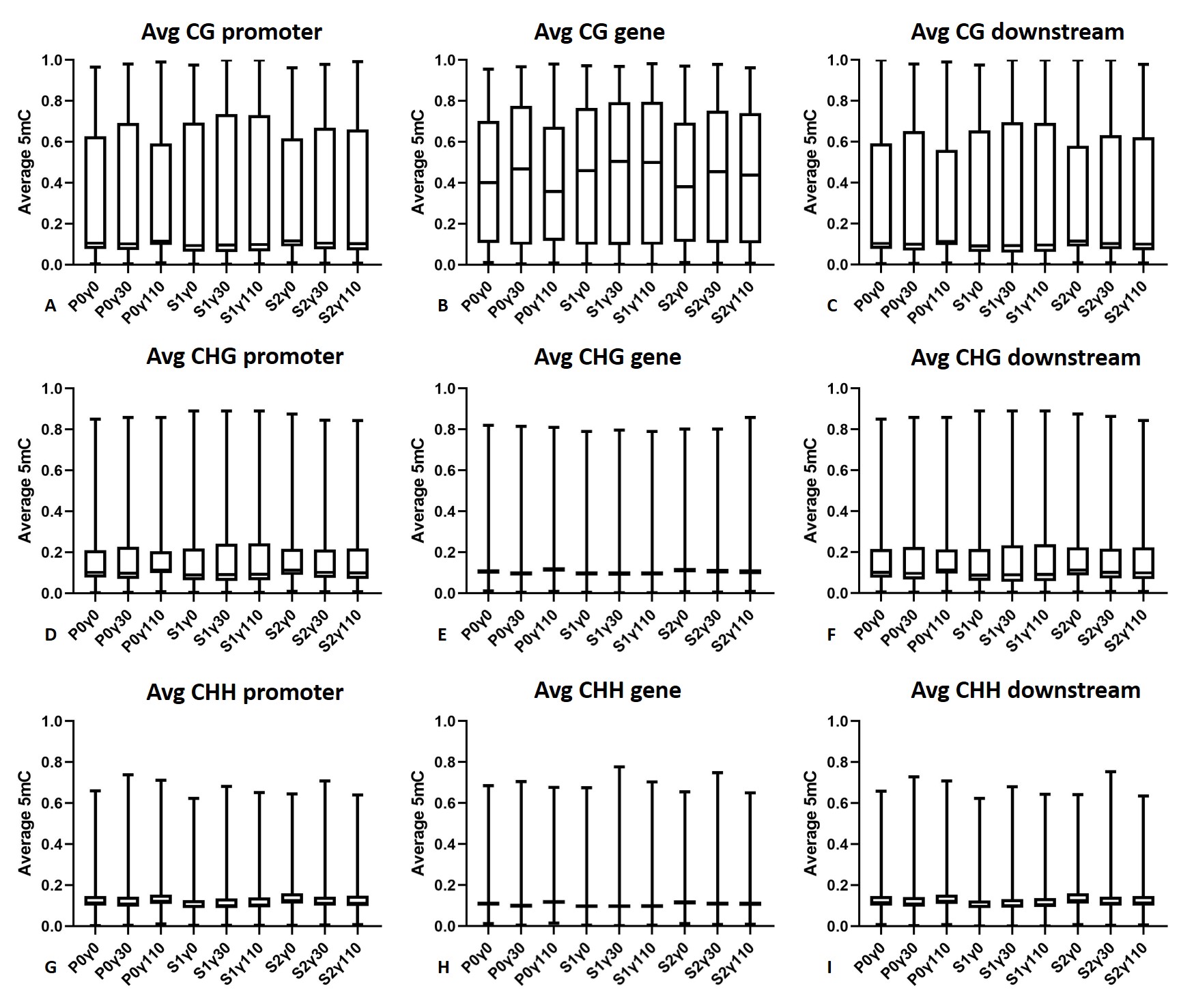
*Proportion of methylated cytosines to total cytosines averaged over all promoter associated regions (2kb upstream of the gene)(A, D, G), genes (B, E, H), and 2kb downstream regions of the genes (C, F, I) for every generation (P0 (Parent generation), S1 (generation 1), S2 (generation 2)) of A. thaliana plants (γ_0_ = control condition (<0.1µGy/h), γ_30_ = 30 mGy/h, γ_110_= 110 mGy/h, the dose rates γ_60_ = 60 mGy/h and γ_430_ = 430 mGy/h were not included in the WGBS) per methylation context (CG: A, B, C; CHG: D, E, F; CHH: G, H, I). Boxplot with median, whiskers indicate minimum and maximum value found in the data set.*

**Supplementary Figure S6:** *Venn diagrams of overlapping genes with differentially methylated regions (DMRs) in their promoter-associated regions over generations (P0 (Parent generation), S1 (generation 1), S2 (generation 2)) in condition* γ_30_ (30 mGy/h) *in the P0*γ_30_ *vs S1*γ_30_*, P0*γ_30_ *vs S2*γ_30_*, and S1*γ_30_ *vs S2*γ_30_ *comparisons. A: Hypomethylated, B: Hypermethylated*


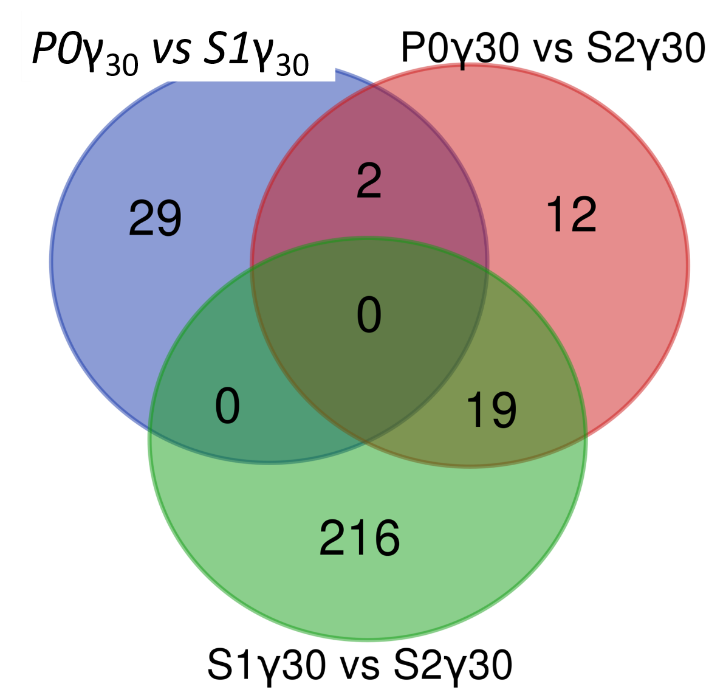

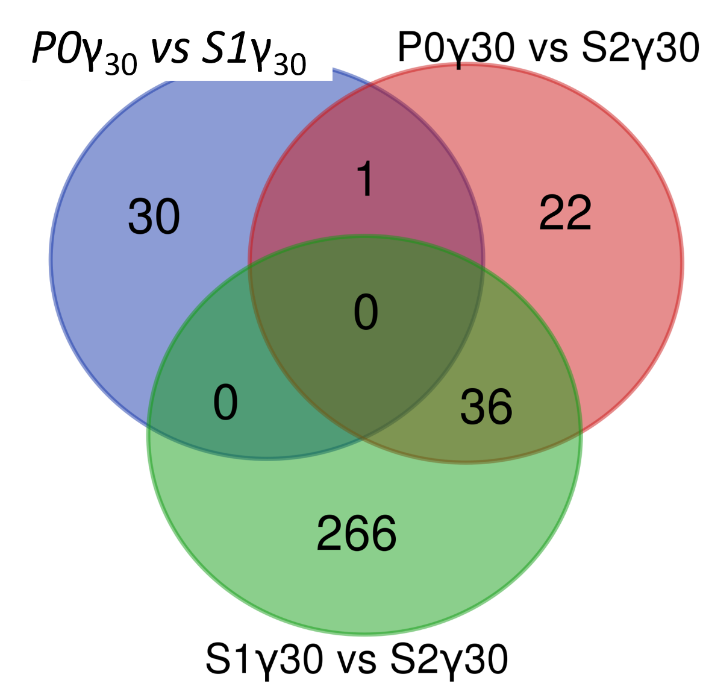


**A. Hypomethylated**

**B. Hypermethylated**

**Supplementary Table S6:** *List of overlapping genes with hypomethylated differentially methylated regions (DMRs) in their promoter-associated regions over generations (P0 (Parent generation), S1 (generation 1), S2 (generation 2)) in condition* γ_30_ (30 mGy/h) *in the P0*γ_30_ *vs S1*γ_30_*, P0*γ_30_ *vs S2*γ_30_*, and S1*γ_30_ *vs S2*γ_30_ *comparisons.*

| **Comparisons** | **Number of genes** | **Genes** |
| --- | --- | --- |
| *P0*γ_30_ *vs S1*γ_30_ *P0*γ_30_ *vs S2*γ_30_ | 2 | LOG7  SCRL5 |
| *P0*γ_30_ *vs S2*γ_30_ *S1*γ_30_ *vs S2*γ_30_ | 19 | AT1G66060  AT1G19240  NPF2.11  AT4G11450  OST1A  ILA  AT5G08670  ARK2  PCMP-H35  MYB82  LECRK13  AT4G13442  ATG18G  AT2G14793  XI-F  AKR4C9  RPS13  EXO70B1  CYP90A1 |

**Supplementary Table S7:** *List of overlapping genes with hypermethylated differentially methylated regions (DMRs) in their promoter-associated regions over generations (P0 (Parent generation), S1 (generation 1), S2 (generation 2)) in condition* γ_30_ (30 mGy/h) *in the P0*γ_30_ *vs S1*γ_30_*, P0*γ_30_ *vs S2*γ_30_*, and S1*γ_30_ *vs S2*γ_30_ *comparisons.*

| **Comparisons** | **Number of genes** | **Genes** |
| --- | --- | --- |
| *P0*γ_30_ *vs S2*γ_30_ *P0*γ_30_ *vs S2*γ_30_ | 1 | AT5G14140 |
| *P0*γ_30_ *vs S2*γ_30_ *S1*γ_30_ *vs S2*γ_30_ | 36 | AT1G21580 SMC5 AT5G47830 AT3G56790 AT5G13760 TBL21 AT4G09587 AT4G33660 WCRKC2 AT1G35500 AT1G61050 SGR2 AT5G35510 MED19A PCMP-E2 AT1G06790 AT5G51580 MAP1D AT2G17305 AT2G13630 AT1G05910 AT4G05586 PME61 AT5G06800 AT4G25510 SPT AT5G02385 MYB108 AT3G27490 AT4G03620 AT3G29120 ALDH2C4 AT5G05830 BHLH130 AT5G05090 AGL52 |

**
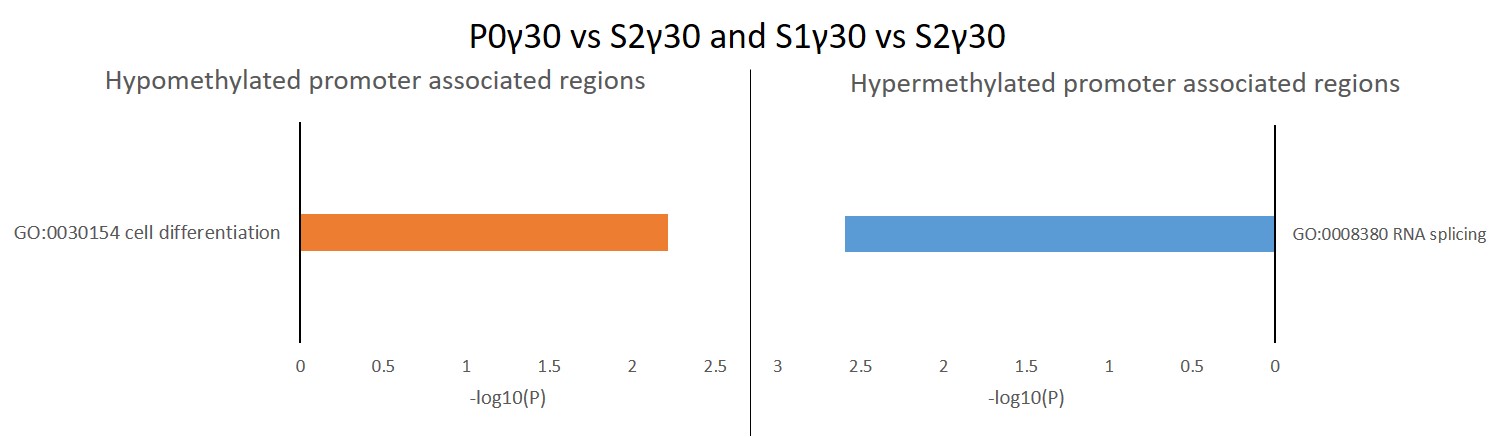
**

**Supplementary *Figure S7:*** *GO term enrichment for hypo- and hypermethylated differentially methylated regions (DMRs) in the promoter-associated regions of Arabidopsis thaliana in the overlap between the comparisons P0γ_30_ vs S2γ_30_* and *S1γ_30_ vs S2γ_30_ (γ_30_ (30 mGy/h), γ_0_ (control condition (<0.1µGy/h)), P0 (Parent generation), S1 (generation 1), S2 (generation 2)). S1 came from a previously exposed generation and S2 came from a line with two previously exposed generations.*


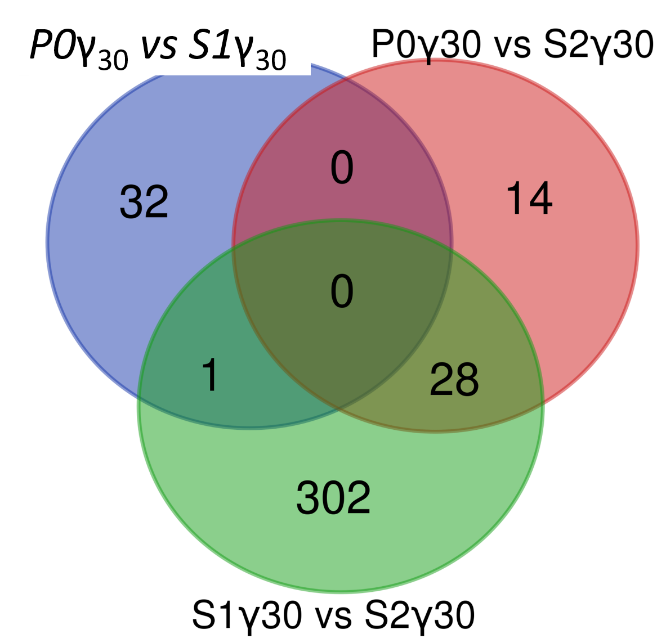

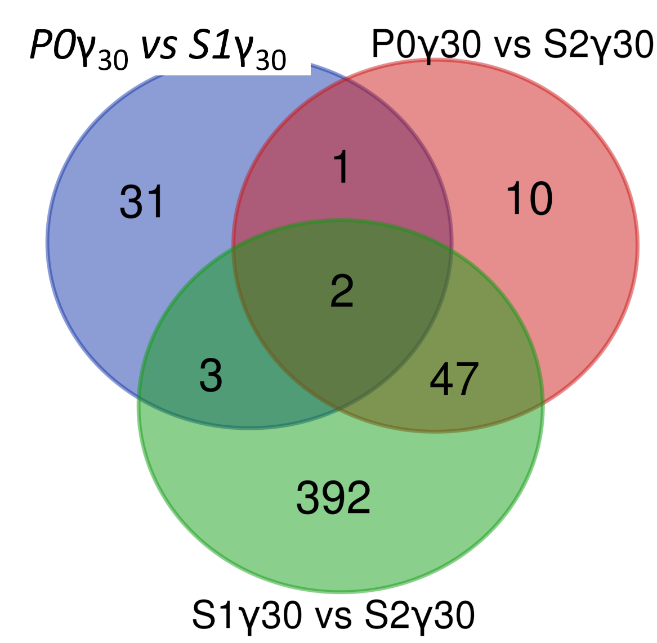


**A. Hypomethylated**

**B. Hypermethylated**

**Supplementary Figure S8:** *Venn diagrams of overlapping genes with differentially methylated regions (DMRs) in their gene bodies over generations (P0 (Parent generation), S1 (generation 1), S2 (generation 2)) in condition* γ_30_ (30 mGy/h) *in the P0*γ_30_ *vs S1*γ_30_*, P0*γ_30_ *vs S2*γ_30_*, and S1*γ_30_ *vs S2*γ_30_ *comparisons. A: Hypomethylated, B: Hypermethylated*

**Supplementary Table S8:** *List of overlapping genes with hypomethylated differentially methylated regions (DMRs) in their gene bodies found over generations (P0 (Parent generation), S1 (generation 1), S2 (generation 2)) in condition* γ_30_ (30 mGy/h) *in the P0*γ_30_ *vs S1*γ_30_*, P0*γ_30_ *vs S2*γ_30_*, and S1*γ_30_ *vs S2*γ_30_ *comparisons.*

| **Comparisons** | **Number of genes** | **Genes** |
| --- | --- | --- |
| *P0*γ_30_ *vs S1*γ_30_ *S1*γ_30_ *vs S2*γ_30_ | 1 | AT2G15860 |
| *P0*γ_30_ *vs S2*γ_30_ *S1*γ_30_ *vs S2*γ_30_ | 28 | AT5G49950  AT5G15680  AT5G03180  HRD1B  ATMYB3R5  FBX5  ARABIDILLO-2  AT1G72420  AGD5  PAS2  RPS15AC  AT5G08630  AT2G47090  RECQL4B  EMB2756  CYP71B10  ITPK4  AT4G38760  FH20  AT3G57370  AT5G53900  XI-F  AT1G56120  AT3G19900  PDF1B  4CLL6  ROS1  SYCO ARATH |

**Supplementary Table S9:** *List of overlapping genes with hypermethylated differentially methylated regions (DMRs) in their gene bodies found over generations (P0 (Parent generation), S1 (generation 1), S2 (generation 2)) in condition* γ_30_ (30 mGy/h) *in the P0*γ_30_ *vs S1*γ_30_*, P0*γ_30_ *vs S2*γ_30_*, and S1*γ_30_ *vs S2*γ_30_ *comparisons.*

| **Comparisons** | **Number of genes** | **Genes** |
| --- | --- | --- |
| *P0*γ_30_ *vs S1*γ_30_ *P0*γ_30_ *vs S2*γ_30_ *S1*γ_30_ *vs S2*γ_30_ | 2 | AT1G10320  VIP4 |
| *P0*γ_30_ *vs S1*γ_30_ *P0*γ_30_ *vs S2*γ_30_ | 1 | VDAC5 |
| *P0*γ_30_ *vs S1*γ_30_ *S1*γ_30_ *vs S2*γ_30_ | 3 | AT1G15130  ACO1  NUA |
| *P0*γ_30_ *vs S2*γ_30_ *S1*γ_30_ *vs S2*γ_30_ | 47 | AT5G46710  APRR7  AT1G55680  AT2G01440  AT4G16800  AT1G79540  AT1G61550  AT5G01660  AT2G45520  AT2G41830  AT2G25320  RPN3A  AT1G61105  SLK2  AT1G51810  AT4G18810  RBOHI  AT1G15290  AT5G11850  TPR16  Phox2  AT2G45700  AT4G18060  AT5G01700  PRP19B  AT1G17930  TIF3F1  AT5G04710  NHX4  BRCA2B  AT4G40000  TAP46  AT5G02340  AT5G02720  ASA1  ASK6  SCAB2  Tudor1  AT5G54520  SIK1  CPL2  WAKL20  AT4G10930  MEE25  ESP  CNGC1  AT4G02660 |

**Supplementary
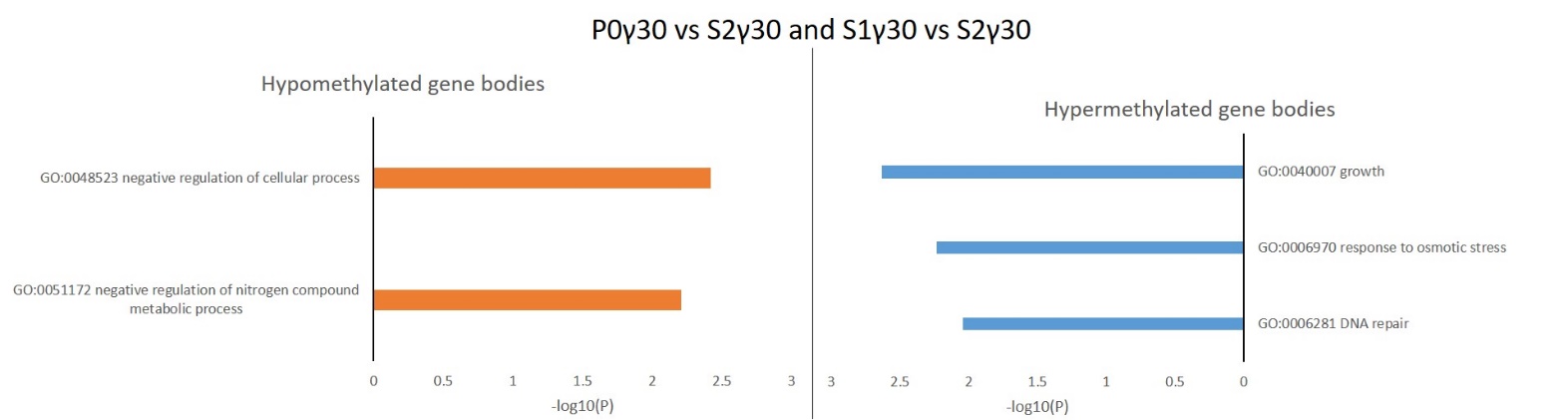
*Figure S9:*** *GO term enrichment for hypo- and hypermethylated differentially methylated regions (DMRs) in the gene bodies of Arabidopsis thaliana in the overlap between the comparisons P0γ_30_ vs S2γ_30_* and *S1γ_30_ vs S2γ_30_ (γ_30_ (30 mGy/h), γ_0_ (control condition (<0.1µGy/h)), P0 (Parent generation), S1 (generation 1), S2 (generation 2)). S1 came from a previously exposed generation and S2 came from a line with two previously exposed generations.*
